# Mechanical single-molecule potentiometers with large switching factors from *ortho*-pentaphenylene foldamers

**DOI:** 10.1038/s41467-020-20311-z

**Published:** 2021-01-08

**Authors:** Jinshi Li, Pingchuan Shen, Shijie Zhen, Chun Tang, Yiling Ye, Dahai Zhou, Wenjing Hong, Zujin Zhao, Ben Zhong Tang

**Affiliations:** 1 State Key Laboratory of Luminescent Materials and Devices, Guangdong Provincial Key Laboratory of Luminescence from Molecular Aggregates, South China University of Technology, 510640 Guangzhou, China; 2 State Key Laboratory of Physical Chemistry of Solid Surfaces, College of Chemistry and Chemical Engineering, Xiamen University, 361005 Xiamen, China; 3 Department of Chemistry, The Hong Kong University of Science and Technology, Clear Water Bay, Kowloon, Hong Kong China

**Keywords:** Electronic devices, Molecular machines and motors, Molecular machines and motors

## Abstract

Molecular potentiometers that can indicate displacement-conductance relationship, and predict and control molecular conductance are of significant importance but rarely developed. Herein, single-molecule potentiometers are designed based on *ortho*-pentaphenylene. The *ortho-*pentaphenylene derivatives with anchoring groups adopt multiple folded conformers and undergo conformational interconversion in solutions. Solvent-sensitive multiple conductance originating from different conformers is recorded by scanning tunneling microscopy break junction technique. These pseudo-elastic folded molecules can be stretched and compressed by mechanical force along with a variable conductance by up to two orders of magnitude, providing an impressively higher switching factor (114) than the reported values (ca. 1~25). The multichannel conductance governed by through-space and through-bond conducting pathways is rationalized as the charge transport mechanism for the folded *ortho*-pentaphenylene derivatives. These findings shed light on exploring robust single-molecule potentiometers based on helical structures, and are conducive to fundamental understanding of charge transport in higher-order helical molecules.

## Abstract (web summary)

Molecular potentiometers that can indicate displacement-conductance relationship, and predict and control molecular conductance are of significant importance but rarely developed. Here, the authors design a robust single-molecule potentiometers based on helical structures and give insight in fundamental understanding of charge transport in higher-order helical molecules.

## Introduction

Advances of miniaturization in electronic devices unveil the possibility of adopting molecular electronics as primary elements in electronic circuity^1^. Plenty of molecules are extensively studied and employed as molecular wires, molecular switches, and molecular transistors^2,3^. Certain special electronic components like potentiometers that can reveal the displacement-conductance relationship, and ultimately predict and control molecular conductance by external mechanical force, are of high importance but rarely developed. The very limited molecular potentiometers^4^ in the literature are mainly created based on linear molecules. The alkanes^5^, silanes^6^, and other linear systems with *cis–trans* isomers are common structures used as mechanical triggered switches because their conductance can be modulated by mechanical force via the deformation of the flexible alkane chains or *cis–trans* isomerization. The switching factor, defined as the ratio of the conductance of the highest conductance state to that of the lowest conductance state, is an important parameter to quantify modulation ratio^5,7^. The most-reported molecules usually have low mechanical switching factors ~10^4–7^. Although it is reported that precise control of tilted angles of molecular wires can lead to a conductance increase of an order of magnitude up to 25-folds^8–10^, these switching factors are still far from satisfactory. Giant variations in molecular conformation, as well as charge transport mechanism, are desired to greatly alter conductance and achieve a controllable conductance–displacement relationship, which is conducive to realizing efficient molecular potentiometers with large switching factors.

Owing to the considerable potential as functional organic nanostructures, *ortho*-phenylenes and their derivatives have been extensively studied in various research fronts^11–13^. They usually adopt a well-defined secondary structure, in which three repeating monomeric phenylene units form a helical turn^14,15^. Moreover, *ortho*-phenylenes often show dynamic conformational interconversion^16–18^ in solutions and exhibit pseudo-elastic properties that allow elongations without permanent deformation^19^. Such kind of backbone flexibility differentiates *ortho*-phenylenes from conventional linear molecules, making it possible to respond sensitively to mechanical stimuli and further control the charge transport behavior by stimulation. The superposition of offset π–π stacking interaction parallel to the helix axis and through-bond conjugation can endow *ortho*-phenylenes with multichannel charge transport in multidimensional degree^20,21^. With these merits, *ortho*-phenylenes have been theoretically predicted as potential candidates for molecular electronics long ago^22^, working as single-molecule solenoids, molecular springs, pressure sensors, and single-molecule potentiometers. However, there is scarcely any research that has literally applied *ortho*-phenylenes as molecular electronics in practice.

**Fig. 1:**
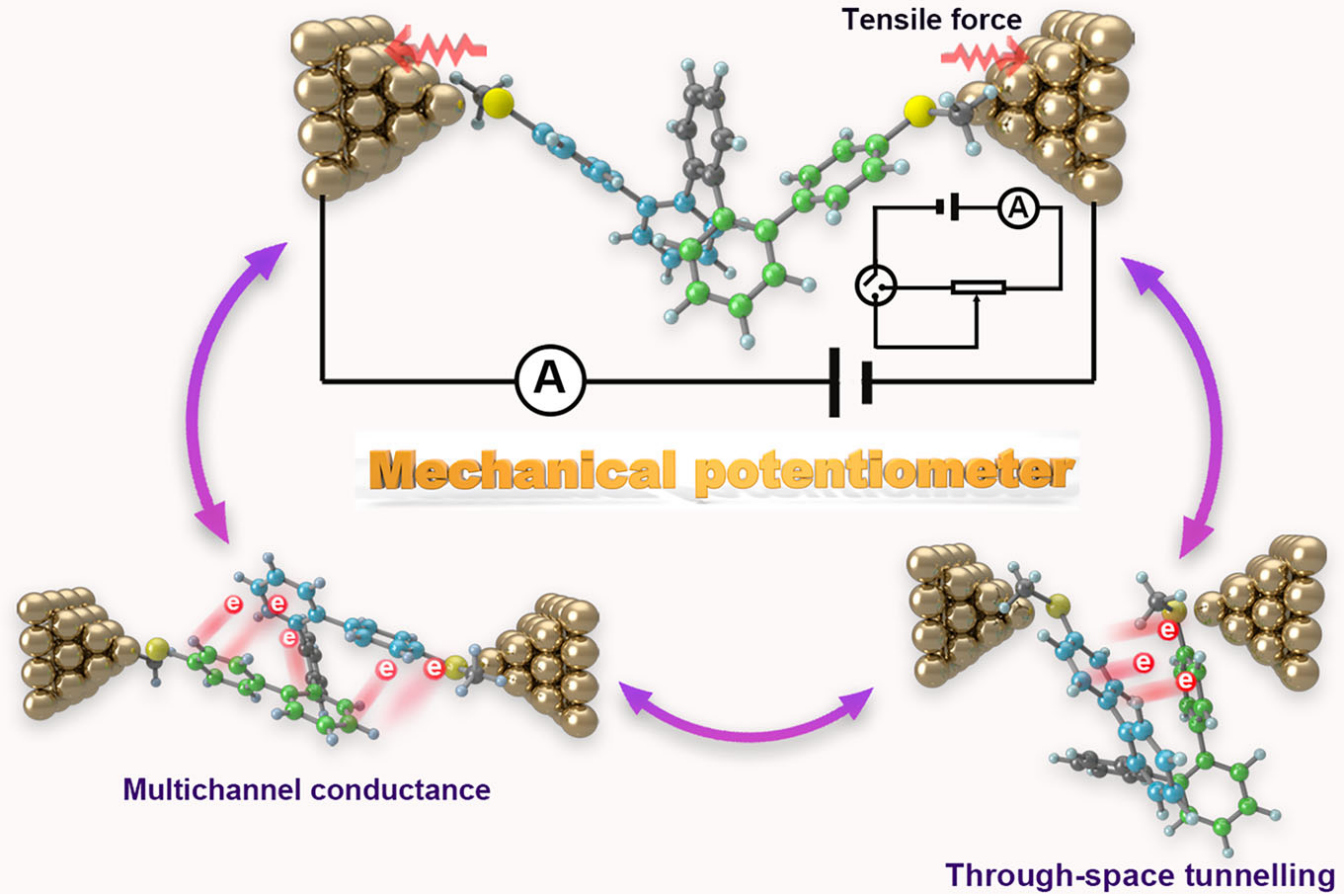
Schematic illustration of a mechanical single-molecule potentiometer based on *o*-PP-2SMe. The tensile force originated from the movement of the Au tip can change the contact conformers of *o*-PP-2SMe then change the conductance of *o*-PP-2SMe aiming to control the circuit current. The change of conformers also leads to the alteration of the charge transport mechanism. The inset is the circuit diagram containing the potentiometer.

*ortho*-Pentaphenylene (*o*-PP) consisting of five *ortho*-position-linked phenyl rings is an intriguing and representative molecule for *ortho*-phenylenes. It holds the helical feature of *ortho*-phenylenes but with relatively less conformers, which makes it the most suitable model to study the optical and electronic characteristics of helical molecules. In this work, we wish to report the feasibility of *ortho-*phenylenes as molecular electronics for the first time based on *o*-PP. These *o*-PP derivatives can undergo conformational interconversion in solutions, rendering different kinds of conformers and thus multiple conductances as revealed by scanning tunneling microscopy break-junction (STM-BJ) technique^23–25^. In addition, their conformers are sensitive to solvents, leading to feasible conductance modulation that has not been found in other systems. More importantly, these pseudo-elastic molecules can be stretched and compressed by the mechanical force of the Au tip along with a dramatically variable conductance by up to two orders of magnitude, demonstrating the great potential as angstrom-scale single-molecule potentiometers (Fig. 1). The flicker noise analysis and theoretical calculation demonstrate the coexistence of through-bond and through-space conducting pathways, furnishing a characteristic of multichannel conductance. These results provide an in-depth insight into the conformational dependence of photoelectric behaviors of *o*-PP derivatives and establish a new avenue toward robust molecular potentiometers.

## Results

### General characterization

**Fig. 2:**
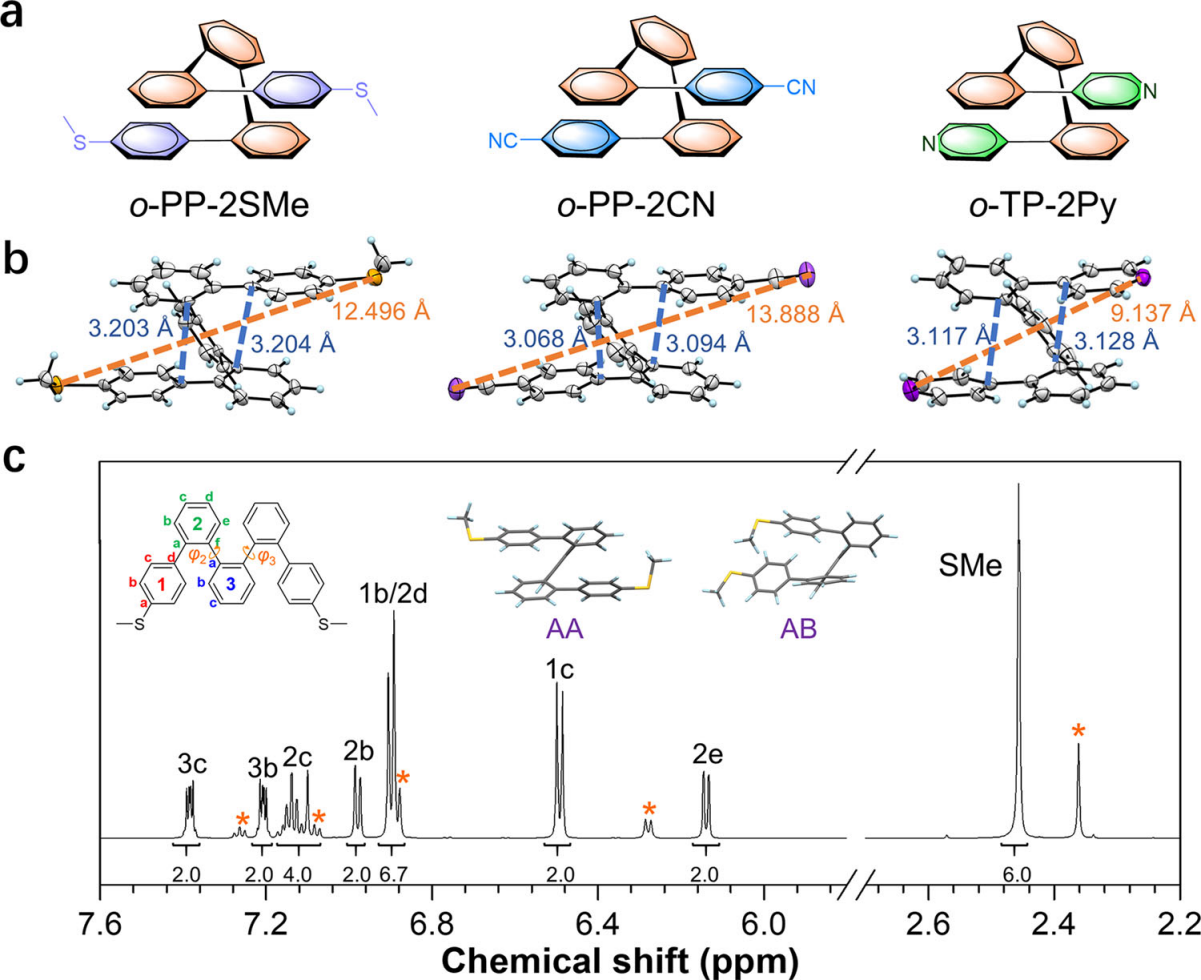
General structural characterization for *o*-PP derivatives. **a** Chemical structures for *o*-PP-2SMe, *o*-PP-2CN, and *o*-TP-2Py, respectively. **b** Single crystals for *o*-PP-2SMe, *o*-PP-2CN, and *o*-TP-2Py, respectively. The orange dashed lines refer to the distances between sulfur atoms with exact values in orange. The blue dashed lines refer to the closest interplane distances between two biaryl groups with exact values in blue. **c** ^1^H NMR spectrum at −5 °C of *o*-PP-2SMe in AA conformer (500 MHz, CD_2_Cl_2_) with major peaks assigned to specific protons of *o*-PP-2SMe in AA conformer. Asterisks: the minor signals from AB conformer. The insets include the schematic structure of *o*-PP-2SMe with certain letters referring to specific protons and numbers referring to the aryl rings, and two distinguishable conformers in the NMR analysis, the AA conformer corresponding to the major signals and the AB conformer corresponding to the minor signals. The two internal dihedral angles *φ*_2_ and *φ*_3_ are also labeled in the inset.

A series of *o*-PP derivatives, *o*-PP-2SMe, *o*-PP-2CN, and *o*-TP-2Py, terminated with different anchoring groups of methylthio (SMe), cyano (CN), and pyridines (Py), respectively, that can interact with Au atom, are designed and constructed with details in Supplementary Information (Supplementary Fig. 1). The crystal structures validate the folded helical structures of all the molecules (Fig. 2a). The unit cell consists of both enantiomeric helices in all crystals (Supplementary Fig. S2), without predisposition toward a specific handedness of the folded structure (Supplementary Figs. 3–5). There is a pair of biaryl groups closely aligned in a face-to-face antiparallel orientation in all three molecules. The shortest interplane distances between two biaryl groups are about 3.1−3.2 Å in three molecules, much shorter than the typical distance (3.5 Å) for the π–π stacking interaction^26–28^, disclosing the existence of intramolecular through-space conjugation. On the other hand, the torsion angles between the central phenyl ring and adjacent phenyl rings are very large (~40°−60°) in these structures (Supplementary Table 1), suggesting that the through-bond conjugation along the molecular backbone is weak.

These molecules undergo conformational interconversion in solutions with low-energy barriers, resulting in multiple conformers, as revealed by NMR analysis. Taking *o*-PP-2SMe as an example, its ^1^H NMR spectrum at −5 °C (Fig. 2b) is the superposition of the contributions from different folding states. The key to define the overall conformational states is the biaryl torsion angles along the molecular backbone, especially the two internal dihedral angles *φ*_2_ and *φ*_3_ as indicated in the inset of Fig. 2b^15^. Based on the scan of the energies of different conformers and further geometry optimization (Supplementary Fig. 20), three kinds of conformers with energy minima are found for *o*-PP-2SMe, which are referred to AA, AB, and BB conformers (Supplementary Fig. 21). The AA conformer holds an antiparallelly folded structure identical to that obtained in crystal. It possesses the lowest energy among all these conformers and accounts for the major signals in ^1^H NMR, which places the characteristic proton (H_2e_) into the shielding zone of π–π stacked phenyl rings and gives rise to distinctive upfield-shifted doublet peaks at ~6.15 ppm^29^. The minor signals are assigned to the co-parallelly folded AB conformer. The 2D exchange spectroscopy (EXSY) exhibits clear cross-peaks and illustrates that the protons giving rise to these minor signals are in exchange with those for major signals (Supplementary Fig. 29), validating that they originate from different conformational states but not impurities. The high-performance liquid chromatography also confirms that *o*-PP-2SMe is of high purity without signals from impurities (Supplementary Fig. 6). The signals for the major AA conformer can be successfully assigned using 2D NMR experiments (COSY, HMQC, and HMBC) (Supplementary Figs. 30–32). Based on the integration of the peaks, the populations of the discernable major AA and minor AB conformers at −5 °C are determined as ~85% and 15%. The BB conformer adopts an elongated “open” helix, but it is undetectable on the NMR timescale because it is of the highest energy and relatively less stable in comparison with the other two conformers. Further, these conformational states are capable of mutual transformation, because the signal peaks in ^1^H NMR spectra become broadened and coalesced at room temperature (Supplementary Figs. 33–35, 40, and 41) and their 2D experiments also verify the coexistence of multiple conformers (Supplementary Figs. 37–39, 42–44). Similar results are also found for *o*-PP-2CN and *o*-TP-2Py from the calculation results (Supplementary Figs. S21 and S22), implying that it is a general character stemming from the rotatable *o*-PP backbone. These results unveil that these molecules experience conformational interconversion among three relatively stable conformers in solution on the NMR timescale. The AA and AB conformers can be directly distinguished in NMR spectra, but the population of BB conformer is too small to be detected by NMR measurement.

The active intramolecular rotation of *o*-PP derivatives will dissipate excited-state energy and thus weaken the photoluminescence (PL)^18,30^. But when these intramolecular rotations are suppressed by spatial constraint in the aggregated state, the nonradiative relaxation of the excited state is suppressed and the radiative decay is strengthened^31^, which will give rise to enhanced PL for these molecules. To validate this hypothesis, the PL behaviors of these molecules in tetrahydrofuran (THF) solutions and aggregates are investigated. In dilute THF solution, they show weak PL peaks at 388‒392 nm (Supplementary Fig. 8), which are consistent with the PL peak of the reported *o*-PP (390 nm)^18^. Low absolute PL quantum yields (*Φ*_PL_s) of 6.6%, 21.6%, and 9.0% are measured for *o*-PP-2SMe, *o*-PP-2CN, and *o*-TP-2Py, respectively. Interestingly, when these molecules form aggregates by adding a large amount of poor solvent water into their THF solutions, the PL intensities are enhanced apparently (Supplementary Fig. 9), presenting a typical aggregation-enhanced emission (AEE) phenomenon^32^. These molecules can also emit stronger PL in solids than in solutions with PL peaks at 384–392 nm. And the *Φ*_PL_s are increased to 14.3%, 34.7%, and 26.8% for *o*-PP-2SMe, *o*-PP-2CN, and *o*-TP-2Py, respectively, which further validate the AEE property. These findings confirm that there are active intramolecular rotations and conformational interconversion in these folded molecules in solutions.

### Multiple conductances

As the regulation of intramolecular rotations endows *o*-PPs with the interesting photophysical property of AEE, it is envisioned that the dynamic conformational interconversion may also bring about intriguing charge transport ability. The scanning tunneling microscopy break-junction (STM-BJ) technique is widely used to study the charge transport behavior at the single-molecule level^33–36^. The conductance of a single-molecule junction highly depends on the contact geometry of the target molecule with the electrodes. To decipher the conformational impact on the molecular conductance, STM-BJ technique is applied to characterize the conductance of these molecules in single-molecule junctions. Typical direct tunneling traces with exponential decay and one-dimensional (1D) histograms shown in Supplementary Fig. 10 verify that there are no conductance plateaus observed in pure solvent experiments, except for an initial conductance feature at *G*_0_ (*G*_0_ = 2*e*^2^/*h*), corresponding to the single Au–Au atomic conductance^37^.

**Fig. 3:**
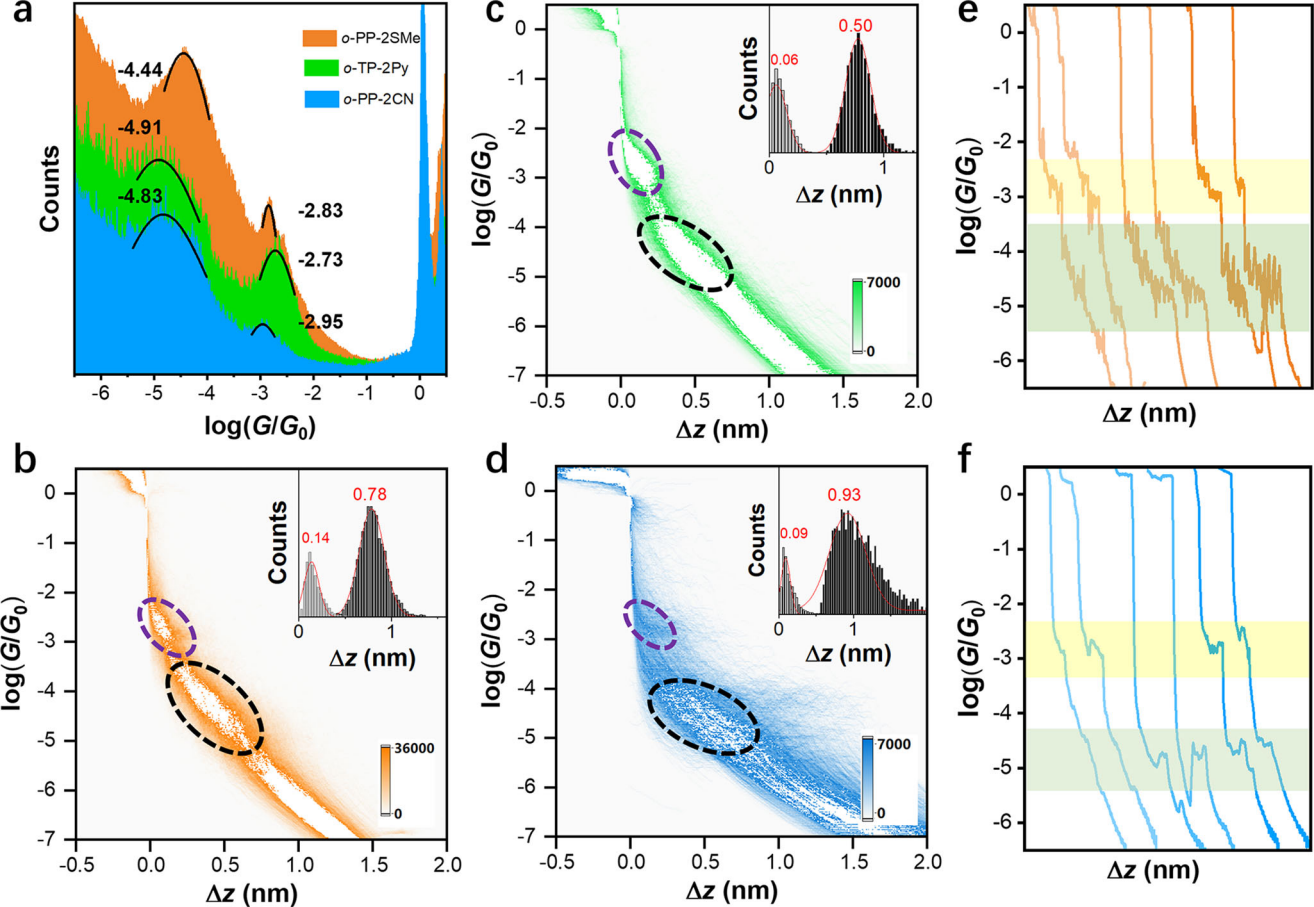
STM-BJ measurements of *o*-PP derivatives in the concentration of 0.2 mM. **a** One-dimensional conductance histograms for *o*-PP-2SMe, *o*-TP-2Py, and *o*-PP-2CN respectively, along with the values of the conductance peaks labeled beside the fitting curves. **b**–**d** Two-dimensional conductance–displacement histograms for *o*-PP-2SMe, *o-*TP-2Py, and *o*-PP-2CN, respectively. The purple dashed circles label the density clouds of HC state, while the black dashed circles label the density clouds of LC states. Insets: the light-gray histograms refer to relative displacement distributions of HC states, while the dark-gray histograms refer to relative displacement distributions of LC states. **e**, **f** Typical individual traces for *o*-PP-2SMe and *o*-PP-2CN, respectively. The yellow frames correspond to the plateau of HC states, while the green frames correspond to the plateau of LC states.

For statistical analysis, the 1D histograms of the molecules (0.2 mM) in the mixed THF/mesitylene (THF/TMB) solvents (1:4, *v*/*v*) are constructed from over 5000 individual conductance–distance traces (Fig. 3a), and the determined conductance is summed up as Supplementary Table 2. Interestingly, all three molecules share a characteristic of multiple conductances in common, indicative of a feature stemming from the aromatic backbones rather than the anchoring groups. The representative individual conductance–displacement traces illustrated in Fig. 3e–f and Supplementary Fig. 11 also depict that a high-conductance (HC) plateau and a low-conductance (LC) plateau occur after the rupture of Au–Au atomic contact at *G*_0_, corresponding to two different molecular junctions. The HC and LC plateaus can appear individually or simultaneously at a single break-junction setup. The corresponding HC peaks for *o*-PP-2SMe, *o*-PP-2CN, and *o*-TP-2Py are located 10^−2.83±0.01^ *G*_0_, 10^−2.95±0.02^ *G*_0_, and 10^−2.73±0.02^ *G*_0_, while the LC peaks are distributed at 10^−4.44±0.03^ *G*_0_, 10^−4.83±0.01^ *G*_0_, and 10^−4.91±0.02^ *G*_0_, respectively, determined from Gaussian fitting. The HC values are higher than the LC ones for *ca*. 40, 75, and 150 times, respectively. It is reasonable to speculate that the multiple conductances originates from the different conformers of these molecules in connection with the two electrodes, and the HC peaks belong to the AB conformers and the LC peaks originate from the AA conformers.

To further confirm the origins of the HC and LC peaks, two-dimensional (2D) conductance–displacement histograms are generated by aligning each conductance trace after the point contact ruptures and overlaying all the conductance traces. As shown in Fig. 3b, *o*-PP-2SMe shows two conductance density clouds circled by dashed lines, in accordance with the HC and LC peaks in the 1D histogram. The stretching distance of the HC state is about 0.14 nm (the inset of Fig. 3b), disclosing that the actual junction length for the HC state is ~0.64 nm after calibration by adding 0.50 nm snap-back distance^38^. The stretching distances are in good agreement with the S–S distances in the AB conformer of *o*-PP-2SMe (Supplementary Table 3). Given the stretching distance of 0.78 nm, the junction length of LC state is about 1.28 nm after calibration, consistent with the S–S distance in the crystal structure of AA conformer. Besides, *o*-PP-2CN and *o*-TP-2Py also exhibit two tilted conductance density clouds in each 2D histogram. The junction lengths of the HC state of *o*-PP-2CN and *o*-TP-2Py are 0.59 and 0.56 nm, respectively, close to the N–N distances in their AB conformers (Supplementary Table 3). The junction lengths of the LC states of *o*-PP-2CN and *o*-TP-2Py are 1.43 and 1.00 nm, respectively, consistent with the distances of N–N bonds in their AA conformers. These evidences prove that these molecules at different conformational states can form varied single-molecule junctions with the electrodes via the two terminated anchoring groups. In general, the conductance values of HC and LC states differ from each other by 1.5–2.0 orders of magnitude, associated with AB and AA conformers, respectively. This distinct conductance difference enables us to gain in-depth insights into the individual charge transport behavior of each conformer at the molecular scale.

### Solvent effect

**Fig. 4:**
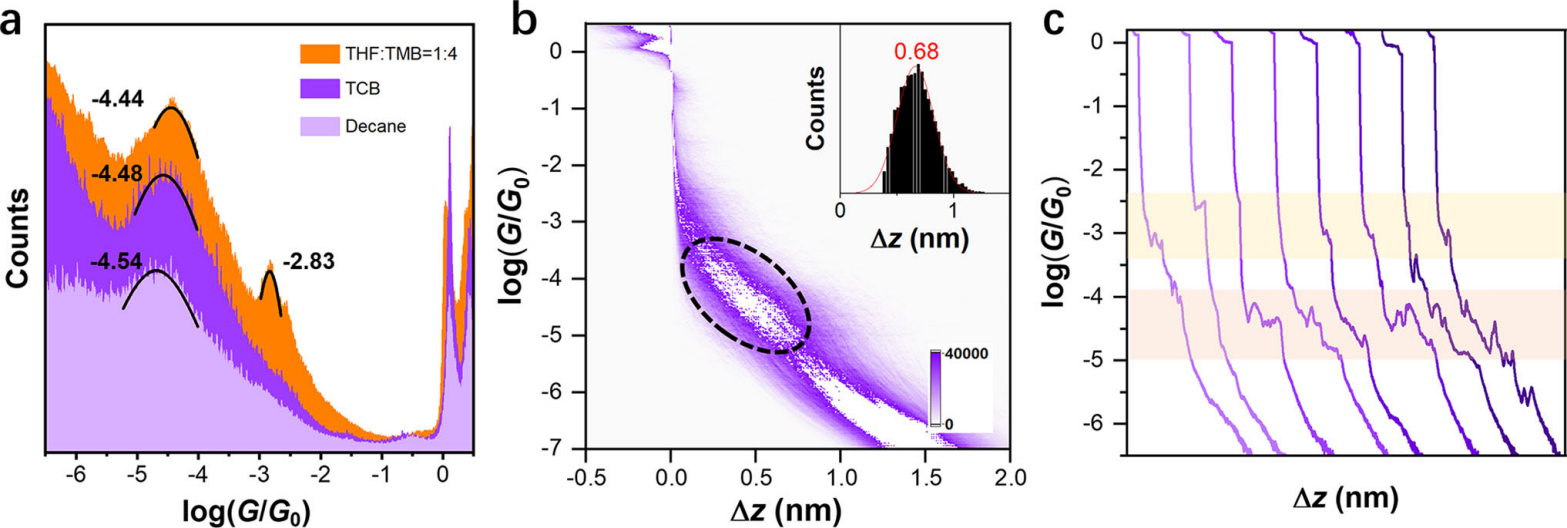
The solvent effect on conductance measurement of *o*-PP-2SMe in the concentration of 0.2 mM. **a** One-dimensional conductance histograms of o-PP-2SMe in different solvents along with the values of the conductance peaks labeled beside the fitting curves. **b** Two-dimensional conductance–displacement histogram of *o*-PP-2SMe in TCB with black dashed circle labeling the density cloud of LC state. Inset: relative displacement distribution. **c** Typical individual traces of *o*-PP-2SMe in TCB. The yellow frame corresponds to the plateau of HC states, while the orange frame corresponds to the plateau of LC states.

The intramolecular stacking of the foldamers that adopt well-defined secondary structures is usually sensitive to the variation of the environmental conditions because the local interaction among repeating units can be easily impacted. To evaluate the conformational response to various environments, the conductance of these molecules in different solvents is investigated. The 1D conductance histograms of *o*-PP-2SMe constructed from over 5000 individual traces in THF/TMB mixture (1:4, *v*/*v*), 1,2,4-trichlorobenzene (TCB), and *n*-decane are performed. Different from the two distinct conductance peaks recorded in THF/TMB mixture, the 1D histograms of *o*-PP-2SMe in TCB and *n*-decane only exhibit one single conductance peak and a tiny shoulder at around 10^−3.0^ *G*_0_ (Fig. 4a). The most probable conductance peaks of *o*-PP-2SMe in TCB and *n*-decane appear at 10^−4.48±0.01^ *G*_0_ and 10^−4.54±0.02^ *G*_0_, respectively, basically consistent with that of the LC state in THF/TMB mixture. Meanwhile, there is only one clear tilted conductance density cloud can be observed in each 2D histogram of *o*-PP-2SMe in TCB and *n*-decane (Fig. 4b and Supplementary Fig. 15). The junction lengths are calculated as 1.18 and 1.24 nm in TCB and *n*-decane after calibration, respectively. In other words, the conductance values obtained in TCB and *n*-decane originate from the antiparallelly folded AA conformer. Typical conductance–displacement traces show that both HC and LC plateaus exist in different measurements in different solvents (Fig. 4c and Supplementary Fig. 14).

In view of that three molecules have no functional groups that can chemically interact with the solvents used in the measurements, the possibility of the chemical reaction between the molecules and the solvents is actually very low. Meanwhile, the interaction between the solvents and the Au atoms are also too weak to affect the contact work function to alter molecular conductance^39,40^. In addition, to exclude the impact from different anchoring groups, the conductance measurements of *o*-TP-2Py and *o*-PP-2CN are also conducted in *n*-decane (Supplementary Figs. 16 and 17 and Table 2). These two molecules share a common characteristic that the HC state of the AB conformer fades while the LC state of the AA conformer remains nearly intact, indicating that the solvent effect on conductance is independent of anchoring groups. The electrostatic solvent–molecule interaction^41,42^ may be the interpretation of the slight conductance decrease of the LC states with the decrease of solvent polarity but can not explain the disappearance of the HC state. So, concerning the polarity difference of the solvents, it is highly possible that these molecules are more prone to adopt AB conformers upon increasing solvent polarity even though the AA conformers are still the major conformers in all kinds of solvents. This behavior is surprisingly different from those of many aromatic foldamers, which favor the more compact, better-folded conformers in more polar solvents because of the decisive solvophobicity^43^.

To further understand this unusual phenomenon, the differences of Gibbs free energy (Δ*E* = *E*_AB_ − *E*_AA_) between AA and AB conformers in *n*-decane (non-polar) and THF (polar) are calculated (Supplementary Table 3), using a solvation model based on density. The Δ*E* is lower in THF than in *n*-decane, indicating that the population of AB conformer is larger in THF. Moreover, AB conformer has a larger dipole moment than AA conformer and thus is more favored in polar solvents (Supplementary Table 3)^21,44^. By comparing the Δ*E*s and dipole moments, these molecules share the same trend that the co-parallelly folded AB conformers are more stable in more polar solvents with little impact from anchoring groups, while the antiparallelly folded AA conformers are always the major conformers with little distributions by polar solvents. So, the distinct conductance variation in different solvents is caused by the conformational alteration of the molecules. These results actually suggest a feasible approach to modulate the conductance of these folded molecules by solvents.

### Mechanical potentiometer

**Fig. 5:**
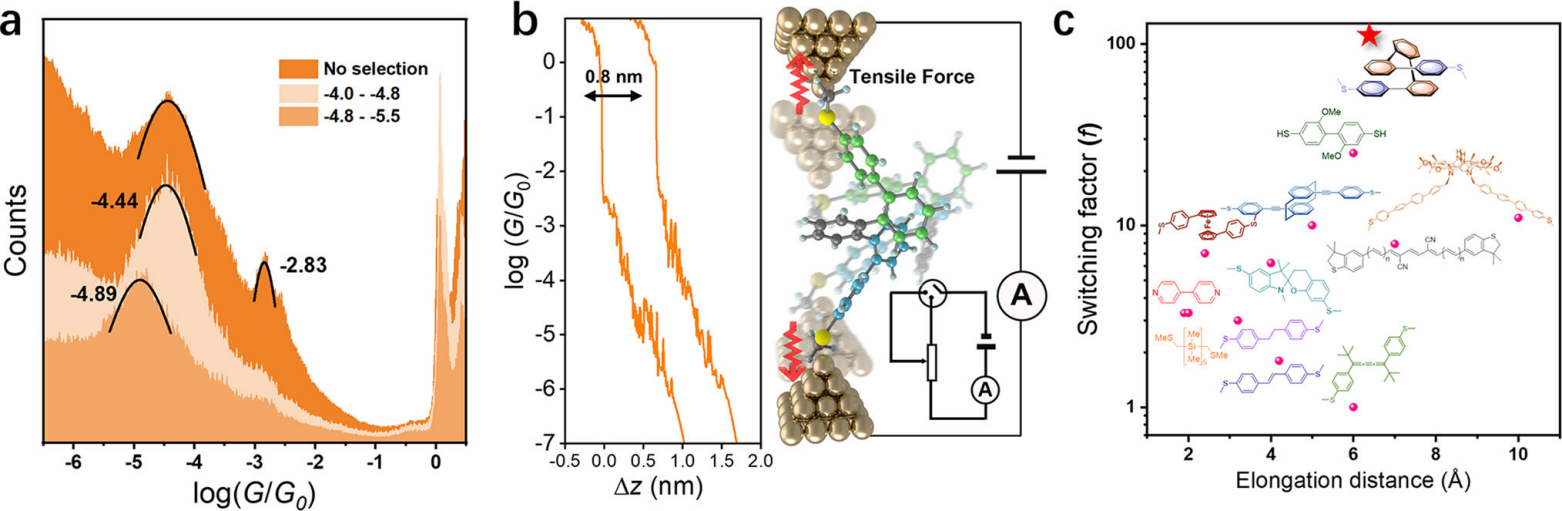
Schematic illustration of *o*-PP derivatives as mechanical potentiometers based on STM-BJ measurement. **a** The one-dimensional histograms for *o*-PP-2SMe without data selection and with data selection among different conductance range in THF: TMB (1:4, *v*/*v*), along with the values of the conductance peaks labeled beside the fitting curves. **b** Typical “downhill” conductance–displacement traces for *o*-PP-2SMe and the schematic illustration of mechanical potentiometer based on *o*-PP-2SMe that can be elongated by tensile force then change the conductance. The inset is the circuit diagram containing the potentiometer. **c** The switching factor (*f*) versus elongation distance for the reported mechanically sensitive molecular wires.

More interestingly, a kind of “downhill” conductance–displacement traces continuously ranging from ~10^−3.0^ *G*_0_ to 10^−6.0^ *G*_0_ are found for *o*-PP-2SMe in STM-BJ measurement (Fig. 5). Similar phenomena have been rarely reported in *meta-* or *para-*isomers^45^. The empty junctions may also exhibit the “downhill” behavior but their lengths are typically <0.5 nm. Given the expected relationship, *G ∼ e*^*-βn*^, the downhill traces (*β* ≈ 0.3 Å^−1^) are distinctly different than empty junctions (tunneling through a vacuum, *β* = 5.5 Å^−1^) in THF/TMB (1:4, *v*/*v*) mixture^4,46^. An additional lower conductance peak at 10^−4.89±0.02^ *G*_0_ can be separated from the LC state (Fig. 5a). These traces indicate that the molecule can undergo a continuous stretching process along with the tensile strength from the lifting of the Au tip (Fig. 5b). Since the molecular backbone is quite flexible and able to convert into different conformers, the *o*-PP-2SMe molecule can be elongated by the Au tip^47^ from a co-parallelly folded AB conformer to a fully stretched BB conformer. This interpretation is corroborated by the fact that downhill conductance traces are also observed for *o*-PP-2CN and *o*-TP-2Py with flexible *ortho*-phenylene backbone but not for the previously reported rigid *para-*isomers with flat conductance plateaus^48,49^. By comparison between the downhill traces and theoretical simulation (Supplementary Fig. 25), it is reasonable to believe that the conductance of BB conformer is around 10^−6^ *G*_0_. The lower conductance of BB conformer than AA conformer is attributed to the poor through-bond conjugation of the highly twisted backbone as well as the absence of through-space conjugation of BB conformer. The additional conductance peak at 10^−4.89±0.02^ *G*_0_ with longer junction length and more obvious 2D density cloud (Supplementary Fig. 14) is assigned to the incompletely stretched conformer between AA and BB conformers as the Au tip is not always able to effectively stretch the molecule to a completely unfolding state. The typical downhill traces and additional lower conductance around 10^−4.90^ *G*_0_ for *o*-PP-2SMe are also found in all three kinds of solvents (Fig. 5c and Supplementary Figs. 15 and 18). This result indicates that *o*-PP-2SMe is flexible and stretchable but prefers the same predominant AA conformer regardless of solvents. The downhill conductance traces are also observed for *o*-PP-2CN and *o*-TP-2Py (Supplementary Figs. 16 and 17) but with a narrower conductance ranging from ~10^−3.0^ *G*_0_ to 10^−5.0^ *G*_0_ without additional conductance peaks. One of the possible reasons is that the coupling between Au and N atom of Py and CN is relatively weaker, which disables the Au tip to fully stretch the molecule before the breaking of junctions^38,50^.

This pseudo-elastic property allowing elongations without permanent deformation addresses the possibility of operating the *o*-PP derivatives as an angstrom-scale helical mechanical potentiometer^5^ as depicted in Fig. 5b. The conformational variable *o*-PP backbone can work as an efficient spring-like resistance device that can be used to continuously control conductance within two orders of magnitude by adjusting the displacement of electrodes. The switching factor of *o*-PP-2SMe is as high as 114 with an elongation distance of 6.4 Å and decay constant of 0.3 Å^−1^, much higher than those of previously reported molecules possessing mechanically triggered conductance variation (*ca*. 1–25) (Fig. 5c)^4–10,46,47,51–53^. It is reported that the *para*-polyphenylenes can have a conductance enhancement of one order of magnitude by changing the tilted angles attaching the gold electrode^8,9^. But our results have shown that *o*-PPs are superior to these traditional linear molecules with more than two orders of magnitude switching factor. Given the interaction between the electrodes and SMe group is not strong enough, it is believed that the switching conductance can be up to three orders of magnitude, the switching factor can reach up to 1000 and the elongation distance can be about 1 nm for the molecular potentiometers based on *o*-PPs with stronger anchoring groups to ensure stronger interaction with electrodes. All these results suggest that *o*-PPs are promising candidates as single-molecule potentiometers with superior conductance variation to linear molecules and precise modulation of conductance by interelectrode gaping.

### Multichannel transmission mechanism

**Fig. 6:**
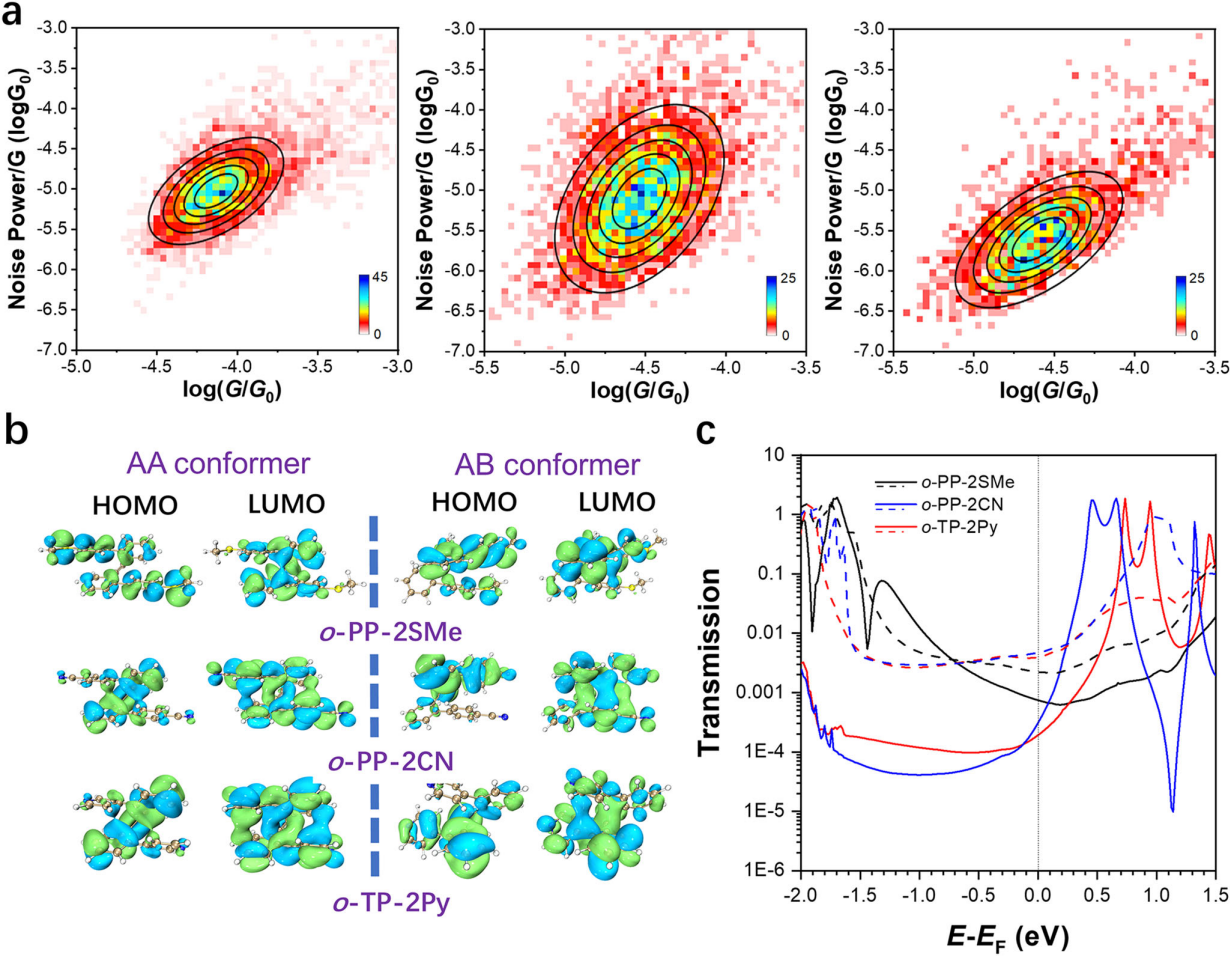
The analyses of multichannel conductance of *o*-PP derivatives based on flicker noise analyses and theoretical calculation. **a** The two-dimensional histograms of normalized noise power versus average conductance for *o*-PP-2SMe, *o*-PP-2CN, and *o*-TP-2Py. **b** Calculated highest occupied molecular orbitals (HOMOs) and lowest unoccupied molecular orbitals (LUMOs) for *o*-PP-2SMe, *o*-PP-2CN, and *o*-TP-2Py in AA and AB conformers. **c** Transmission spectra for *o*-PP-2SMe, *o*-PP-2CN, and *o*-TP-2Py in AA and AB conformers. The solid lines in transmission spectra correspond to AA conformers and the dash lines correspond to AB conformers.

To explore the charge transport pathways in these folded molecules, flicker noise analyses^54^ are carried out. All the flicker noise analyses are focused on the LC state stemming from the major AA conformers in molecular junctions. In the light of previous reports^54,55^, the noise power scaling as *G*^2.0^ is indicative of through-space coupling while *G*^1.0^ stands for through-bond coupling (Supplementary Fig. 19). As shown in Fig. 6a and Supplementary Fig. 19, the noise power of the AA conformers of *o*-PP-2SMe, *o*-PP-2CN, and *o*-TP-2Py scale as *G*^1.8^, *G*^1.9^, and *G*^1.9^, respectively, indicating that through-space and through-bond electronic couplings synergistically participate in the charge transport. It is well-known that the frontier orbitals dominate the molecular conductance^56^. The electron density clouds^57^ shown in Fig. 6b demonstrate that the through-space interaction occurs in the lowest unoccupied molecular orbitals (LUMOs), suggesting charges can travel through eigenchannels opened by non-covalent bonding alignment (through-space channel) like the π‒π stacked dimer molecular junctions^58–61^. Therefore, the flicker noise analysis evidences the dominant through-space coupling that can compensate the weakened through-bond pathway, and contribute significantly to superior conductance to their linear *para*-pentaphenylene (*p*-PP) isomers with a sole through-bond pathway^48,49^.

As the noise power does not completely scale as *G*^2.0^, it is possible that these folded molecules hold through-bond channel in addition to through-space channel for charge transport^62–64^. In order to validate this hypothesis and shed light on the transmission mechanism of these molecules, density functional theory (DFT) simulation is carried out using the ATK software package with nonequilibrium Green’s functions (NEGF). The zero-bias transmission spectra (Fig. 6c) of all three molecules are calculated in their fully relaxed geometries corresponding to AA and AB conformers (Supplementary Figs. 26 and 27). In AA conformers, the calculated conductance of *o*-PP-2SMe is 2.4 and 3.8 times larger than those of *o*-PP-2CN and *o*-TP-2Py (Supplementary Table 4), respectively, which are consistent with the experimental data. The transmission spectra suggest that the main transmission channel for *o*-PP-2SMe is the highest occupied molecular orbital (HOMO), whereas the LUMOs dominate the conductance of *o*-TP-2Py and *o*-PP-2CN, which is in accordance with other reported polyphenylenes with the same anchoring groups^56^. The main cause of the different conductance between *o*-PP-2SMe with electron-donating SMe anchors and the other two molecules with electron-withdrawing CN and Py anchors is the different dominant molecular orbitals. Since the through-space conjugation mainly occurs in LUMOs (Fig. 6b), the through-space pathways provide larger contributions to *o*-PP-2CN and *o*-TP-2Py, resulting in slightly larger noise power scales than *o*-PP-2SMe. The higher conductance of *o*-PP-2CN is ascribed to the closer LUMO to the Fermi level (*E*_F_) than *o*-TP-2Py. On the other hand, in AB conformers, three molecules show similar transmission spectra with close conductance at zero bias. The calculated conductance of the AB conformers (Supplementary Table S4) is ~1.5, 2.3, and 2.2 order of magnitude larger than the AA conformers for *o*-PP-2SMe, *o*-PP-2CN, and *o*-TP-2Py, respectively, which are in consistent with the experimental results.

**Fig. 7:**
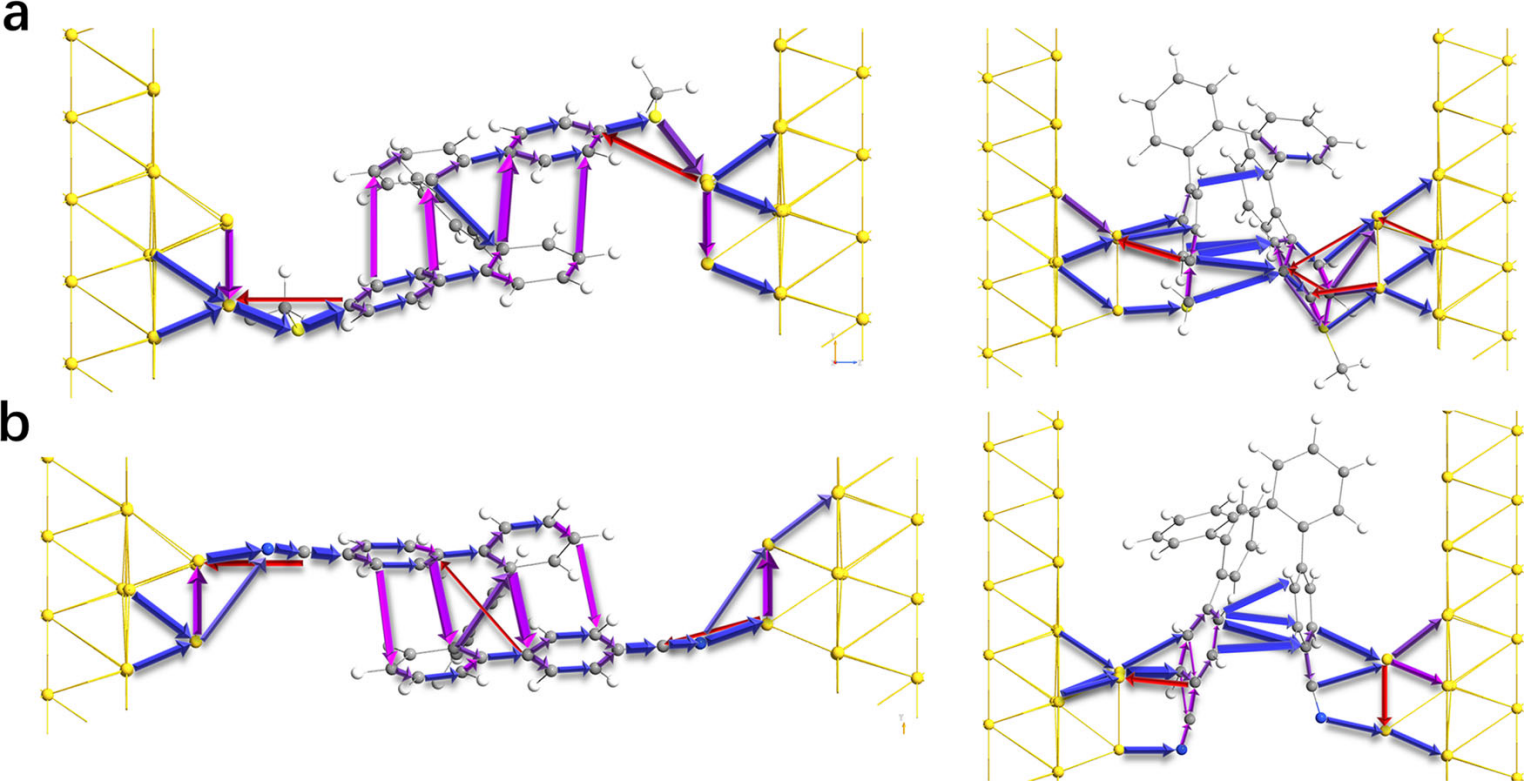
Transmission pathways of *o*-PP derivatives in different conformers. **a** Transmission pathways for the AA and AB conformers of *o*-PP-2SMe. **b** Transmission pathways for the AA and AB conformers of *o*-PP-2CN. The blue arrows represent charge transport from the source electrode to the drain electrode, and the red ones represent the transport in the opposite direction; the larger size of arrows also indicates the higher possibility of charge transport.

The interatomic transmission pathways^65^ further present the directions of charge transport (Fig. 7 and Supplementary Fig. 28). The blue arrows represent charge transport from the source electrode to the drain electrode, and the red ones represent the transport in the opposite direction; the larger size of arrows also indicates the higher possibility of charge transport. On one hand, in AA conformers, through-bond pathways are constructed along the twisted molecular backbones of all three molecules. However, more significantly, effective through-space pathways through the closely stacked aromatic rings are also formed. The simulation of charge transport is in accordance with the flicker noise analysis, demonstrating eminent multichannel conductance in multidimensional degrees. On the other hand, the stimulated charge transport pathways for the AB conformers unveil that the charges prefer to tunnel straight through the through-space pathway between two terminated aromatic rings rather than travel through the entire molecular backbones, consistent with the through-space conjugation feature of intense π-electron clouds overlapping between two closely stacked aromatic rings in these AB conformers (Fig. 6b, Supplementary Figs. 22–24). These findings inspire us to believe that charges can travel efficiently through the through-space conjugated, long, and highly helical molecular backbone. Considering the effective conjugation length of *ortho-*phenylenes is up to eight repeat units^20^, it is promising to further explore robust molecular potentiometers with even larger modulation ratios based on longer *ortho*-phenylenes or molecular coils with high conductance based on long-range through-space conjugation.

## Discussion

In summary, a series of tailor-made *o*-PP derivatives are synthesized and characterized. All the molecules adopt the thermodynamically favorable AA conformers with antiparallelly folded structures in crystals. But in solutions, these molecules undergo conformational interconversion and, in addition to the major AA conformers, co-parallelly folded AB conformers exist as the minor constituents, which are distinguishable in NMR spectra. These intramolecular motions also result in interesting AEE properties for the molecules. Owing to conformational variation, multiple conductances is observed for all the molecules, in which the HC state originates from the AB conformers, while the LC state belongs to the AA conformers. The conductance is also responsive to the alternation of solvent polarity. The AA conformers can transport charges effectively in no need of a polar environment, which is helpful in avoiding higher background noise^66^. Although the AA conformers possess weak through-bond conjugation because of the highly twisted molecular backbones, their conductance is much superior to that of linear *p-*PP isomer, demonstrating the effective compensating conductance stemming from through-space coupling between closely stacked aromatic rings. The flicker noise analysis and theoretical calculation further confirm the coexistence of through-bond and through-space pathways for charge transport, rendering a prominent characteristic of multichannel conductance, which is conducive to achieving high conductance as well as a mechanistic understanding of charge transport in higher-order helical molecules.

More importantly, these pseudo-elastic folded molecules can be stretched and compressed by the mechanical force of the Au tip along with a dramatically variable conductance by up to two orders of magnitude. To the best of our knowledge, the switching factor of *o*-PP-2SMe (114) is the highest value even reported, indicating the great potential as single-molecule potentiometers. The fact that *o*-PPs have larger continuous conductance ranges than conventional alkanes and *para*-phenylenes breaks the stereotype that polyphenylenes are not suitable as a molecular potentiometer. Actually, the helical molecules could be more promising as molecular potentiometers than conventional linear molecules. By strengthening the connection between the electrodes and anchoring groups of *ortho-*phenylenes derivatives with sufficient lengths, high-performance molecular potentiometers with controllable conductance and giant switching factors are expectable.

## Methods

### General characterization

All the chemicals and reagents were purchased from a commercial source and used as received without further purification. ^1^H and ^13^C NMR spectra were measured on a Bruker AV 500 (500 MHz) spectrometer in CD_2_Cl_2_ with tetramethylsilane (TMS, *δ* = 0). High-resolution mass spectra (HRMS) were recorded on a GCT premier CAB048 mass spectrometer operating in MALDI-TOF mode. Single-crystal X-ray diffraction intensity data were collected on a Bruker–Nonices Smart Apex CCD diffractometer with graphite monochromated MoKα radiation. Processing of the intensity data was carried out using the SAINT and SADABS routines, and the structure and refinement were conducted using the SHELTL suite of X-ray programs (version 6.10). UV–vis absorption spectrum was measured on a Shimadzu UV-2600 spectrophotometer. PL spectra were recorded on a Horiba Fluoromax-4 spectrofluorometer. Solution fluorescence quantum yields were measured using a Hamamatsu absolute PL quantum yield spectrometer C11347 Quantaurus_QY. High-performance liquid chromatography spectra were measured using Waters alliance e2695 separation module.

### STM-BJ measurement

In the STM-BJ experiment, 5 μL of the solutions of target molecules (0.2 mM) was directly dropped on the gold substrate, which was used for further STM-BJ experiment. During the measurement, the gold tip is first controlled by a stepper motor (Harmonic Drive, LA-30B-10-F) to get to the approximate position to contact the substrate (<1 μm), thereafter the tip is controlled by a piezo stack (Thorlabs, PC4FL; max drive voltage, 150 V; displacement, 4.6 µm ± 15%) with the voltage applied from 0 to 10 V, with the approach/retract at the speed of 10 nm/s. There is also a feedback system in the setup, when the voltage changing of the piezo stack from 0 to 10 V can not contact gold tips and substrate or break the junction to the detecting limit of our amplifier (~1 pA), the motor would take control of the movement of the tips to offset the distances between tips and substrates. During the repeating breaking and re-connecting operation, the bias is kept at 100 mV, and the real-time conductance is recorded by the home-built I-V converter with a sampling rate of 20 kHz.

### Calibration of the snap-back distance and stretching rate

Conductance measurement in the pure solvent is used for the calibration of the snap-back distance and stretching rate as reported. Because of the existence of snap-back distance, the distance we measured in experiments is the relative distance ∆*z* = *z* − *z*_corr_. Snap-back distance *z*_corr_ refers to the instantly increased distance caused by surface atoms reorganization between two gold electrodes. According to the tunneling equation *G* = *G*_0_ *e*^−*β*^_T_^*z*^, where *β*_T_ is the tunneling decay constant and z is the absolute distance between two electrodes, the tunneling equation can be transferred to log(*G*/*G*_0_) = − 0.434*β*_T_∆*z* − 0.434*β*_T_∆*z*_corr_. Then a plot of log(*G/G*_0_) versus ∆*z* has a slope of −0.434*β*_T_ and an intercept of −0.434*β*_T_∆z_corr_. The decay constant log(∆*G*/*G*_0_)/∆*z* = −5.5 Å^−1^ is chosen to scale the traces in pure solvent. By calibrating the distance from 10^−3.0^ *G*_0_ to 10^−5.0^ *G*_0_ to be 0.36 nm, the stretching rate is determined and used for the calibration of the relative stretching distance. The snap-back distance *z*_corr_ is determined to be 0.5 nm. Calibration of snap-back distance and the stretching rate leads to the absolute stretching distance of molecular junctions *z* = ∆*z* + *z*_corr_, where ∆*z* is calculated from 10^−0.3^ *G*_0_ to one order magnitude lower than the most probable conductance value.

### Data selection process

For each molecule, over 5000 curves are collected and analyzed to form the one-dimensional histograms and two-dimensional conductance–displacement cloud maps from which information concerning the molecular conductance and plateau length can be extracted. An automated process for finding STM-BJ traces with certain molecular plateaus is carried out using Labview processed programs.

To rule out the direct tunneling traces with exponentially decayed conductance, the program will not overlay the traces of which junction lengths is shorter than 0.36 nm in the range from 10^−3.0^ *G*_0_ to 10^−5.0^ *G*_0_ based on calibration. Only the traces with certain molecular plateaus are overlaid to form one-dimensional and two-dimensional histograms.

The formation probability is counted after extracting the traces with certain selected peaks (HC/LC). The formation probabilities are counted as 84.6% (4826/5703), 40.6%, and 37.8% for the LC state of *o*-PP-2SMe, *o*-PP-2CN, and *o*-TP-2Py, respectively. For the HC state, the formation probabilities are counted as 41.4% (2362/5703), 30.8% (1719/5583), and 63.20% (4469/7071) for *o*-PP-2SMe, *o*-PP-2CN, and *o*-TP-2Py, respectively. In addition, there are 10226 out of 11452 (89.3%) traces and 9114 out of 9979 (91.3%) traces are selected to construct the conductance histograms of *o*-PP-2SMe in *n*-decane and TCB, respectively. In the experiment of *o*-PP-2CN and *o*-TP-2Py in *n*-decane, 2285 out of 7130 (35.5%) traces and 5145 out of 12706 (40.5%) traces are selected, respectively.

### Flicker noise measurement

Flicker noise analysis can distinguish the pattern of coupling by noise distribution. The molecular elongation is paused for 150 ms if the junction signal is detected at a bias voltage of 0.1 V and a sampling rate of 20 kHz. The conductance plateau is cut out for discrete Fourier transformation, in which the data are squared to get the noise power density spectra. The data integrated from 100 Hz to 1000 Hz then build the 2D histograms of normalized noise power (Noise Power/*G*) versus the average conductance. According to the previous reports, the noise power of tunneling junction scales as *G*^2.0^ is contributed to complete through-space coupling while *G*^1.0^ refers to complete through-bond coupling.

Two-dimensional Gaussian distribution fitting to each two-dimensional histogram is applied to determine the scaling power, and the black circles represent the contour lines of the fitted Gaussian distribution equation. To figure out the smallest absolute value of the correlation coefficient in the fitted Gaussian distribution equations as the correlation power, the normalized conductance power from *G*^1.0^ to *G*^2.0^ with a 0.1 gradient is successively increased.

### Theoretical calculation

Geometry optimization calculations were carried out with Gaussian 16 program using density functional theory (DFT) and time-dependent density functional theory (TD-DFT) for the ground-state and long-range corrected functional M06-2X applied with 6-31G(d,p) basis set, treating crystal structures as the initial configurations. The vibrations of compounds also calculated under the same method. The flexible scan is processed under the M06-2X/def2-TZVP method, the stable values of *φ*_2_ and *φ*_3_ can be divided into two enantiomeric species: −54°/+126° and +54°/−126°. For simplicity, we illustrate the conformational change of *o*-PP-2SMe in terms of the −54°/+126° set, labeling the −54° state as “A” and the +126° state as “B”. The electron energy of compounds is obtained with M06-2X applied with def2TVZP basis set in both isolated state and solvents (THF and *n*-decane) that using the SMD model. The Gibbs free energy in solvents is calculated using the M06-2X/6-31G(d) method. The electron density clouds are drawn by Multifwn.

### Transmission calculation

All the molecules are optimized based on the procedure described above, and making sure there is no imaginary frequency existed in the finally adopted structures. The compounds then inserted between the Au electrodes to build up model devices by the Atomistic Tool Kit (ATK) software. The molecular junction was formed by the semi-finite left and right electrodes (LE and RE, respectively) and a scattering region where the molecule was settled. A scattering region also comprises screening layers of LE and RE. The molecular device was examined using the Au (111) electrodes (both LE and RE). The Au electrodes were settled to a 5 × 6 × 3 supercell. First, isolated organic molecules were optimized. After the optimization, organic molecules were inserted between the LE and RE, and the distance between the LE and RE was relaxed until the total energy reached a minimum. Next, constraining the Au electrodes, organic molecules were relaxed. During the relaxation, force tolerance was set to 0.05 eV/Å. The distance between Au and S is 2.5 ± 0.1 Å. Then, first-principles calculations were carried out to expose the electronic transport properties. In calculations, the exchange-correlation potential was approximated within the generalized gradient approximation (GGA) with Perdew–BurkeErnzerhof (PBE) functional (GGA.PBE)^14^, for the exchange and correlation effects of the electrons. Double-ζ plus polarization (DZP) basis set for the molecule and single-ζ plus polarization (SZP) basis set for the electrodes. For geometry optimization, 1 k-point for *x*-, *y* directions, and 50 k-points for *z* direction, for transmission spectra and transmission pathways calculation, 5 k-point for *x*-, *y* directions, and 100 k-points for *z* direction. We resolve the interatomic transmission pathways at the Fermi energy with ATK software based on the calculated transmission spectra. A minimum threshold of 10% of the total transmission is used for the pathways.

## Supplementary information

Supplementary Information

## Data Availability

All other data are available from the corresponding author upon reasonable requests. The X-ray crystallographic coordinates for structures reported in this study have been deposited at the Cambridge Crystallographic Data Centre (CCDC), under deposition numbers 2000283 (*o-*PP-2SMe), 2000316 (*o-*PP-2CN), and 2006669 (*o-*TP-2Py). These data can be obtained free of charge from The Cambridge Crystallographic Data Centre via www.ccdc.cam.ac.uk/data_request/cif. Source data are provided with this paper.

## Source data

Source Data

## Code availability

The source code of the algorithms is available for research uses at https://github.com/JinshiLi/oPP-codes Computational data used to arrive at the conclusions presented in the manuscript are available upon reasonable request.
