# Supplementary material for: Mechanical single-molecule potentiometers with large switching factors from ortho-pentaphenylene foldamers
Source: Nat Commun. 2021 Jan 8;12:167. doi: 10.1038/s41467-020-20311-z (PMC7794330; doi:10.1038/s41467-020-20311-z)
Supplement: Supplementary file 1 — Supplementary Information [file 41467_2020_20311_MOESM1_ESM.pdf]

SUPPLEMENTARY INFORMATION

Mechanical single-molecule potentiometers with large switching factors from *ortho*-pentaphenylene foldamers

Jinshi Li,[1,4] Pingchuan Shen,[1,4] Shijie Zhen,[1] Chun Tang,[2] Yiling Ye,[2] Dahai Zhou,[2] Wenjing Hong,[2,]** Zujin Zhao,[1,]* and Ben Zhong Tang[1,3]

[1] *State Key Laboratory of Luminescent Materials and Devices, Guangdong Provincial Key Laboratory of Luminescence from Molecular Aggregates, South China University of Technology, Guangzhou, 510640, China. Email: mszjzhao@scut.edu.cn.*

[2] *State Key Laboratory of Physical Chemistry of Solid Surfaces, College of Chemistry and Chemical Engineering, Xiamen University, Xiamen, 361005, China. Email: whong@xmu.edu.cn.*

[3] *Department of Chemistry, The Hong Kong University of Science and Technology, Clear Water Bay, Kowloon, Hong Kong, China*

[4] *These authors contributed equally to this work.*

Table of Contents

Section 1. Synthesis and characterization........S2
Section 2. X-ray crystallography........S3
Section 4. Photophysical properties........S8
Section 5. Conductance measurements........S9
Section 6. Theoretical calculation........S14
Section 7. NMR spectra........S19

Section 1. Synthesis and characterization

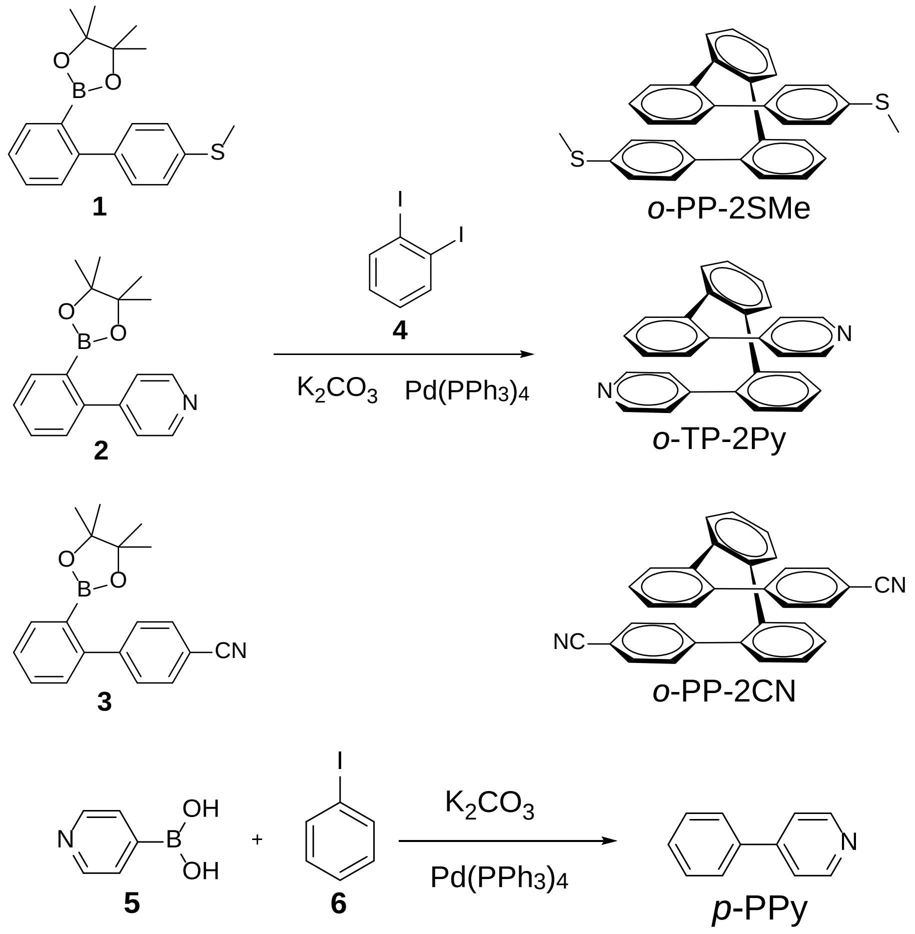

Supplementary Figure 1. Synthesis routes for the *ortho*-phenylene derivatives.

4,4''''-Bis(methylthio)-1,1':2',1'':2'',1''':2''',1''''-quinquephenyl (*o*-PP-2SMe): Into 100 mL two-necked round bottom flask was placed **1** (1.63 g, 3 mmol), **4** (197 mg ,0.6 mmol), $Pd(PPh_3)_4$ (70 mg, 0.06 mmol) and K_2CO_3 (497 mg, 3.6mmol). The flask was evacuated under vacuum and flushed with dry nitrogen three times and then 50 mL mixture of methylbenzene, ethyl alcohol and deionized water (3/1/1, v/v/v) was added. The reaction mixture was heated and refluxed at 95 °C for 16 h. After cooling to room temperature, the mixture was poured into water and extracted with dichloromethane three times. The combined organic layers were dried over angydrous magnesium sulfate. After filtration and solvent evaporation, the crude product was purified by silica-gel column chromatography with mixed petroleum ether and dichloromethane as eluent. White solid of product *o*-PP-2SMe was obtained in 58% yield. ^{1}H NMR (500 MHz, CD_2Cl_2), δ (ppm) : 7.40 –7.35 (m, 2H), 7.23–7.15 (m, 2H), 7.17–7.05 (m, 4H), 6.99 (d, J = 7.0 Hz, 2H), 6.94–6.88 (m, 6H), 6.52 (d, J = 8.0 Hz, 2H), 6.16 (d, J = 8.0 Hz, 2H). ^{13}C NMR (125

MHz, CD_2Cl_2), δ (ppm): 141.94, 140.69, 140.65, 139.37, 137.47, 133.73, 132.91, 131.57, 130.75, 130.22, 128.61, 138.38, 128.05, 127.54, 127.19, 126.84, 17.12. HRMS (MALDI-TOF): *m/z* [M^+] calcd. $C_{32}H_{26}S_2$, 474.1476; found, 474.1478).

[1,1':2',1'':2'',1''':2''',1''''-Quinquephenyl]-4,4''''-dicarbonitrile (*o*-PP-2CN): The procedure was analogous to that described for *o*-PP-2SMe. White solid of *o*-PP-2CN was obtained in 53% yield. ^{1}H NMR (500 MHz, CD_2Cl_2), δ (ppm): 7.50–7.42 (m, 2H), 7.30 (d, J = 8.4 Hz, 4H), 7.27–7.24 (m, 2H), 7.24–7.17 (m, 4H), 6.64 (d, J = 8.5 Hz, 4H), 6.13 (d, J = 7.5 Hz, 2H). ^{13}C NMR (125 MHz, CD_2Cl_2), δ (ppm): 146.15, 140.14, 139.71, 138.65, 132.17, 132.13, 131.90, 129.79, 129.62, 128.97, 128.61, 127.58, 119.54, 110.03. HRMS (MALDI-TOF): *m/z* [M^+] calcd. $C_{32}H_{20}N_2$, 432.1626; found, 432.1618).

2,2''-Di(pyridin-4-yl)-1,1':2',1''-terphenyl (*o*-TP-2Py): The procedure was analogous to that described for *o*-PP-2SMe. White solid of *o*-PP-2Py was obtained in 47% yield. ^{1}H NMR (500 MHz, CD_2Cl_2), δ (ppm): 8.92 (d, J = 5.0 Hz, 4H), 7.52–7.42 (m, 2H), 7.32–7.25 (m, 2H), 7.25–7.18 (m, 2H), 7.06–6.95 (m, 4H), 6.52–6.40 (d, J = 5.0 Hz, 4H), 6.23–6.13 (d, J = 7.0 Hz, 2H). ^{13}C NMR (125 MHz, CD_2Cl_2), δ (ppm): 149.31, 148.97, 140.23, 139.67, 137.67, 137.96, 132.28, 132.07, 129.30, 128.50, 127.58, 124.05. HRMS (MALDI-TOF): *m/z* [M^+] calcd. $C_{28}H_{20}N_2$, 384.1426; found, 384.1436).

4-Phenylpyridine (*p*-PPy): The procedure was analogous to that described for *o*-PP-2SMe. White solid of *p*-PPy was obtained in 96% yield. ^{1}H NMR (500 MHz, CD_2Cl_2), δ (ppm): 8.65 (dd, J = 4.6, 1.6 Hz, 2H), 7.65–7.58 (m, 2H), 7.51–7.38 (m, 5H). ^{13}C NMR (125 MHz, CD_2Cl_2), δ (ppm): 151.57, 148.40, 139.47, 130.37, 128.27, 122.84. HRMS (MALDI-TOF): *m/z* [M^+] calcd. $C_{11}H_{11}N$, 155.0735; found, 155.0727).

Section 2. X-ray crystallography

Crystal data for *o*-PP-2SMe (CCDC 2000283): $C_{32}H_{26}S_2$, *M*w = 474.65, triclinic, P-1, a = 9.6357(10), b = 10.6121(13), c = 12.6923(15) Å, α = 82.645(4)°, β = 78.544(4)°, γ = 77.796(4)°, V = 1238.2(2) Å^3, Z = 2, Dc = 1.273 g cm^{-3}, μ = 0.0234 mm^{-1}, F(000) = 500, T = 171(2) K, R_1 (I > 2σ(I)) = 0.0657, wR_2 (I > 2σ(I)) = 0.0864, R_1 (all data) = 0.1560, wR_2 (all data) = 0.1057.

Crystal data for *o*-PP-2CN (CCDC 2000316): $C_{32}H_{20}N_2$, *M*w = 432.50, orthorhombic, Pca2(1), a =

15.2643(10), b = 13.1444(6), c = 23.4027(15) Å, α = 90°, β = 90°, γ = 90°, V = 4695.5(5) $Å^3$, Z = 8, Dc = 1.224 g cm^{-3}, μ = 0.072 mm^{-1}, F(000) = 1808, T = 172(2) K, R_1 (I > 2σ(I)) = 0.0513, wR_2 (I > 2σ(I)) = 0.1087, R_1 (all data) = 0.1137, wR_2 (all data) = 0.1454.

Crystal data for o-TP-2Py (CCDC 2006669): $C_{28}H_{20}N_2$, Mw = 384.46, monoclinic, Cc, a = 11.4313(4), b = 25.1275(9), c = 8.6287 (3) Å, α = 90°, β = 124.91(10)°, γ = 90°, V = 2032.5(13) $Å^3$, Z = 4, Dc = 1.256 g cm^{-3}, μ = 0.074 mm^{-1}, F(000) = 808, T = 173(2) K, R_1 (I > 2σ(I)) = 0.0361, wR_2 (I > 2σ(I)) = 0.0817, R_1 (all data) = 0.0453, wR_2 (all data) =0.0885.

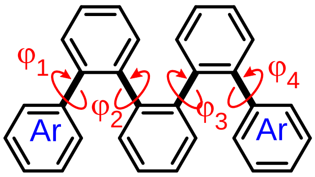

Supplementary Table 1. The torsional angle in the crystal structures of *o*-PP derivatives.

	φ_1 (deg)	φ_2 (deg)	φ_3 (deg)	φ_4 (deg)
***o*-PP-2SMe**	47.23	61.08	59.87	44.54
***o*-PP-2CN**	45.52	51.83	53.21	43.24
***o*-TP-2Py**	37.05	56.07	56.77	46.13

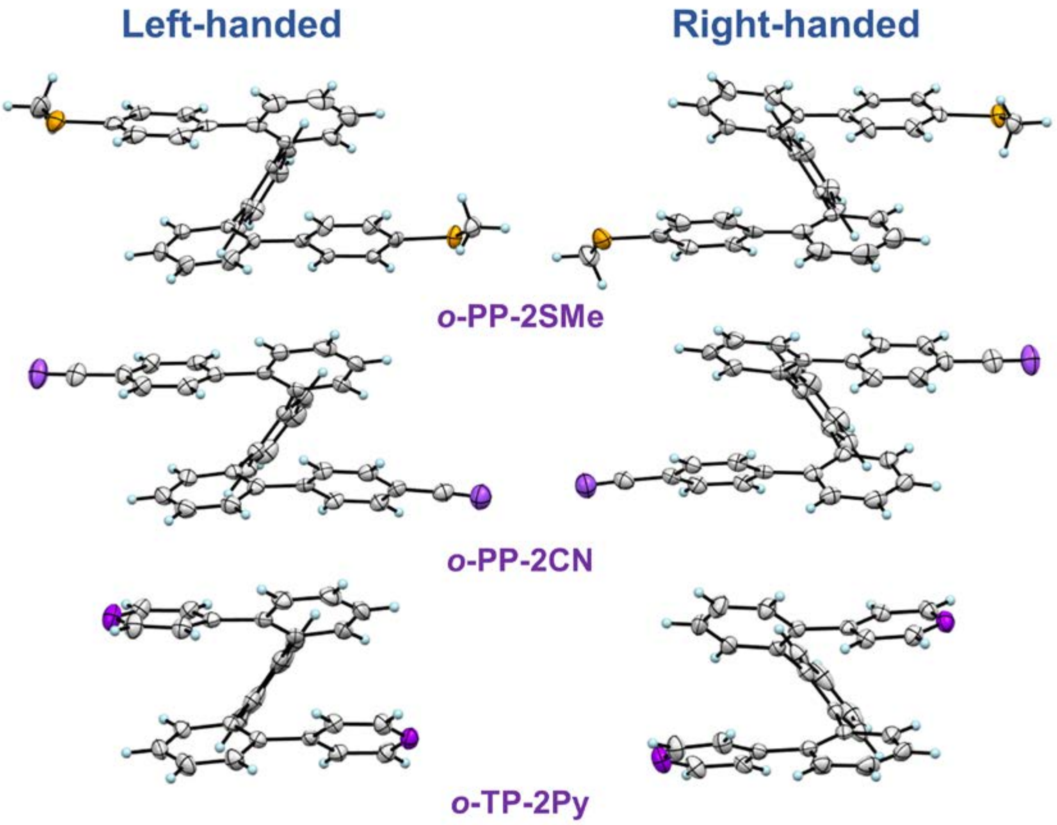

Supplementary Figure 2. Enantiomeric crystal structures of *o*-PP-2SMe, *o*-PP-2CN and *o*-TP-2Py.

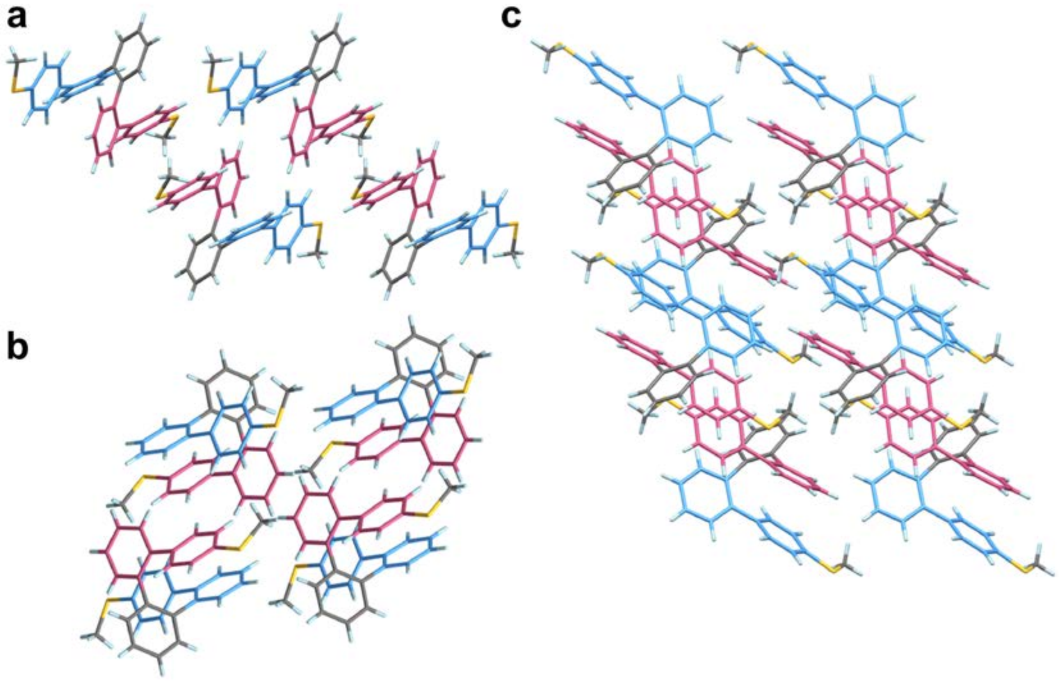

Supplementary Figure 3. Single crystal structure for *o*-PP-2SMe viewed along *a*-axis (A), *b*-axis (B) and *c*-axis (C).

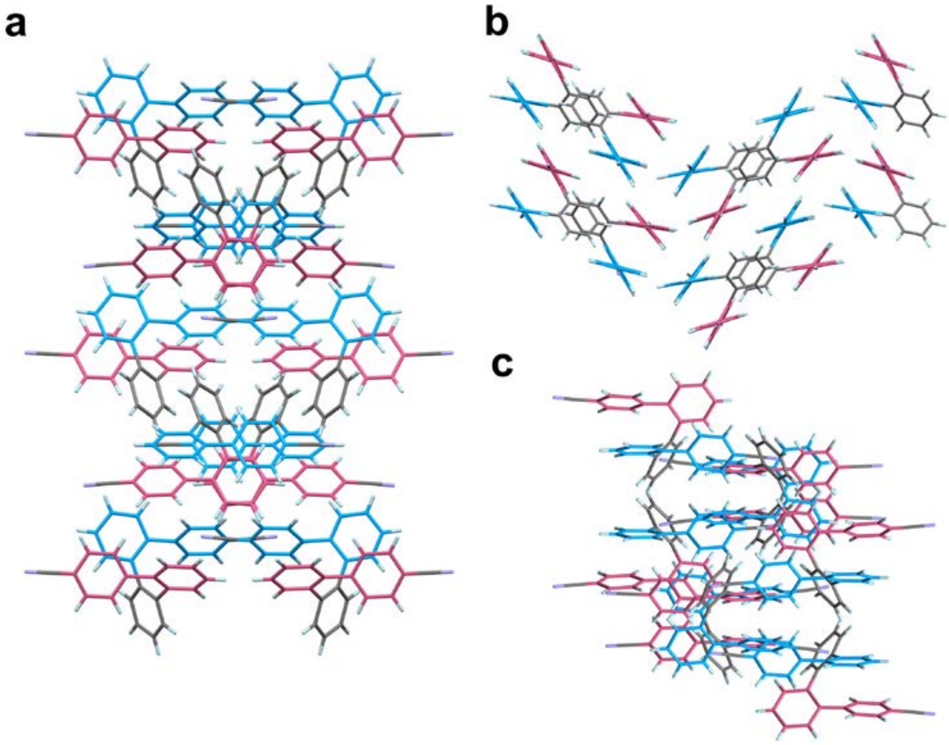

Supplementary Figure 4. Single crystal structure for *o*-PP-2CN viewed along *a*-axis (A), *b*-axis (B) and *c*-axis (C).

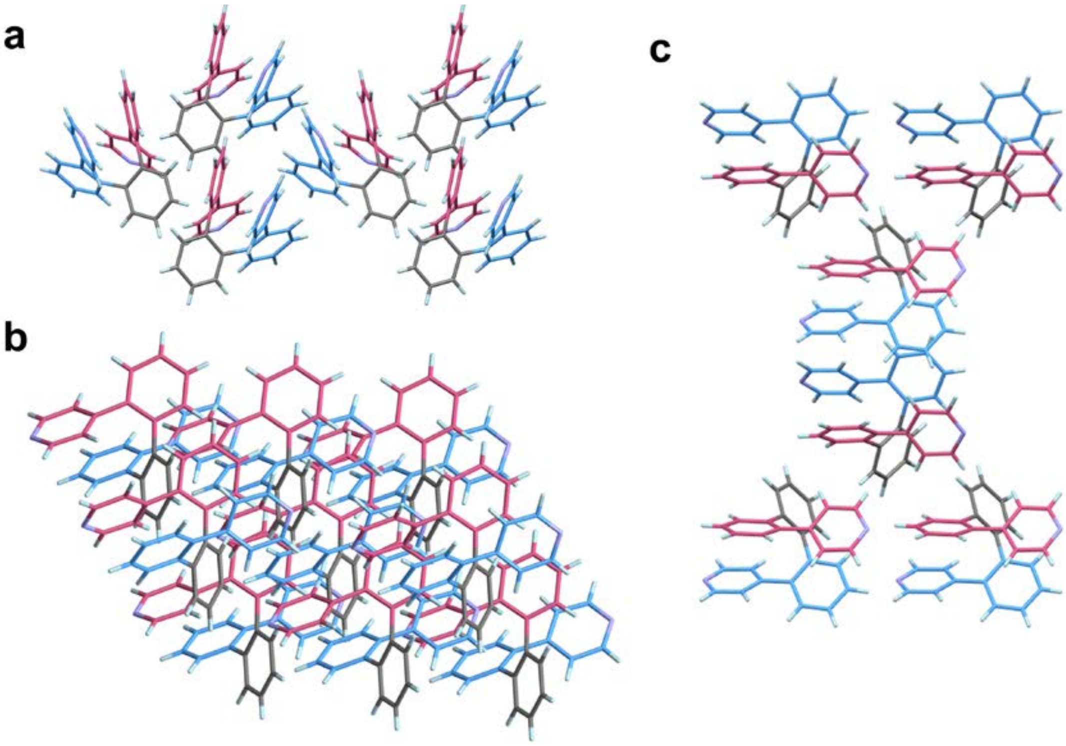

Supplementary Figure 5. Single crystal structure for *o*-TP-2Py viewed along *a*-axis (A), *b*-axis (B) and *c*-axis (C).

Section 3. High performance liquid chromatography

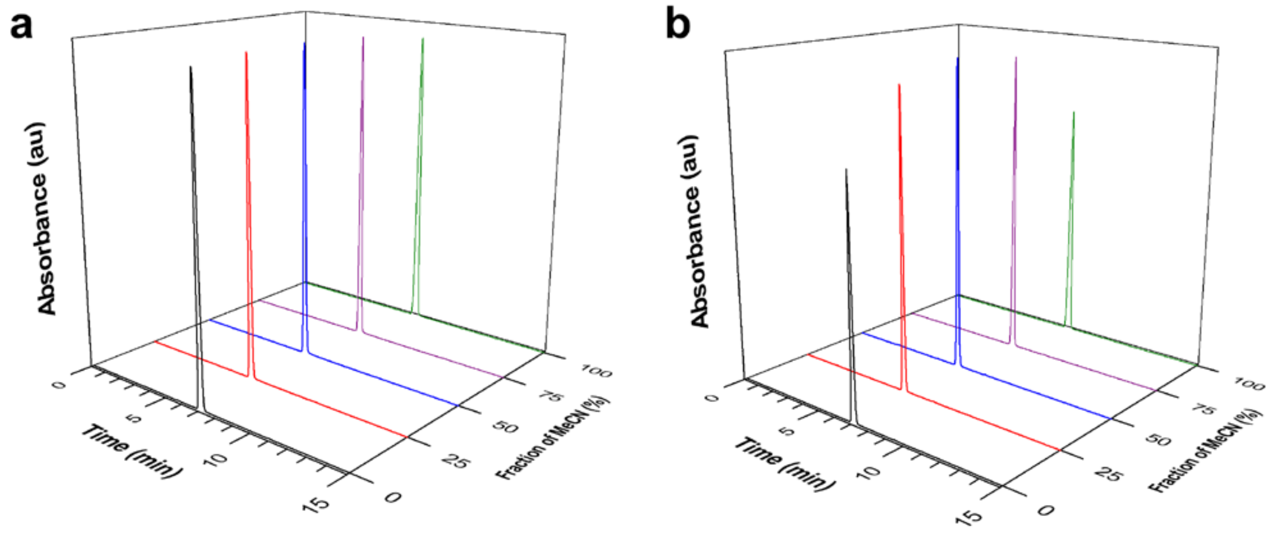

Supplementary Figure 6. **Purity analysis of *o*-PP-2SMe.** The HPLC spectra for *o*-PP-2SMe in MeCN/MeOH mixed solution with different MeCN fractions under **a** 254 nm and **b** 320 nm.

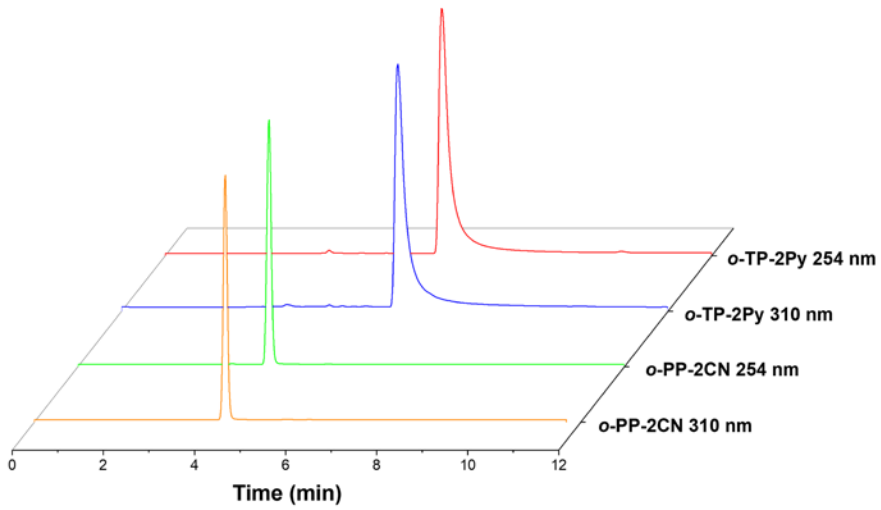

Supplementary Figure 7. **Purity analysis of *o*-PP-2CN and *o*-TP-2Py.** The HPLC spectra for *o*-TP-2Py and *o*-PP-2CN in MeCN solution.

Section 4. Photophysical properties

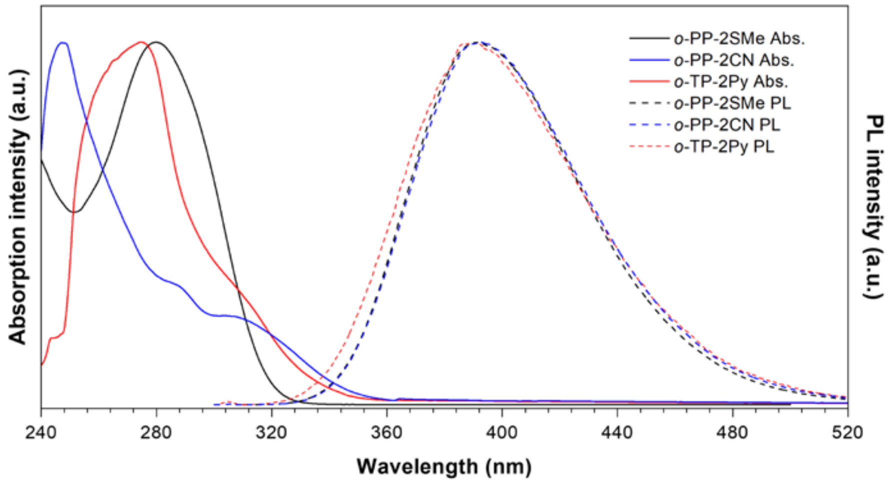

Supplementary Figure 8. **Photophysical properties of *o*-PP derivatives.** Normalized absorption (the solid lines) and photoluminescence (PL, the dash lines) spectra for *o*-PP-2SMe, *o*-PP-2CN and *o*-TP-2Py (λ_{ex} = 280 nm, 10^{-5} M).

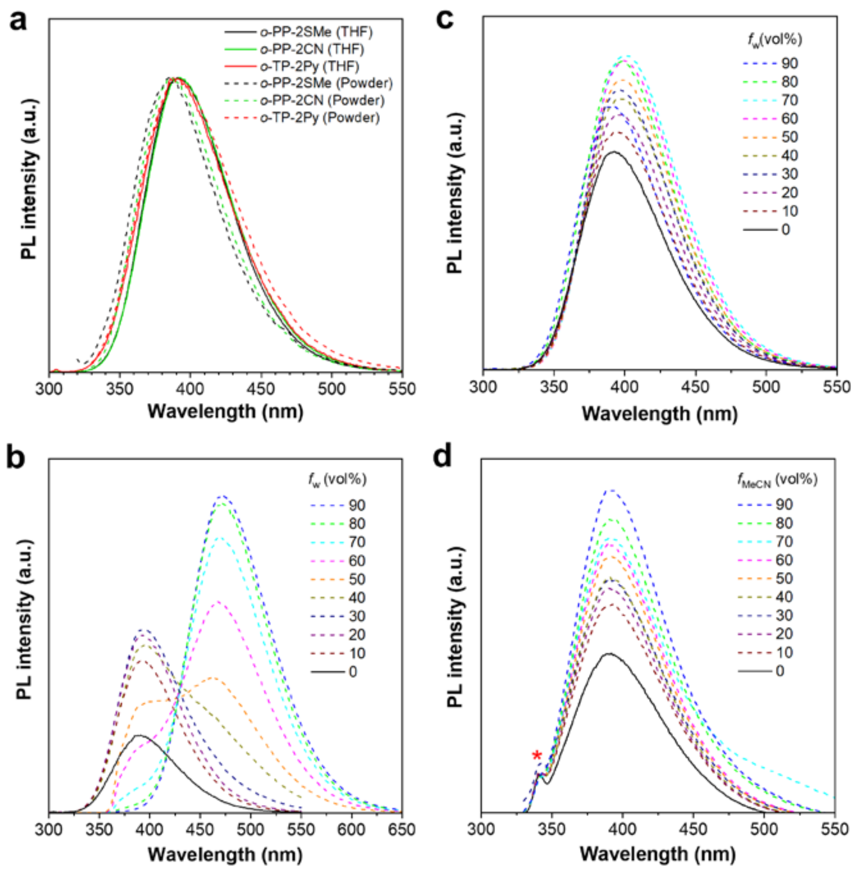

Supplementary Figure 9. **Luminescent performance of *o*-PP derivatives. a** PL spectra for *o*-PP-2SMe, *o*-PP-2CN and *o*-TP-2Py in THF (solid lines) and in solid state (dash lines). PL spectra for **b** *o*-PP-2SMe and **c** *o*-PP-2CN in THF-water mixtures with different water fractions (f_w). **d** PL spectra for *o*-TP-2Py in THF-MeCN mixtures with different MeCN fractions (f_{MeCN}) without the interference of protonation on pyridine. λ_{ex} = 280 nm; concentration = 10^{-5} M.

Section 5. Conductance measurements

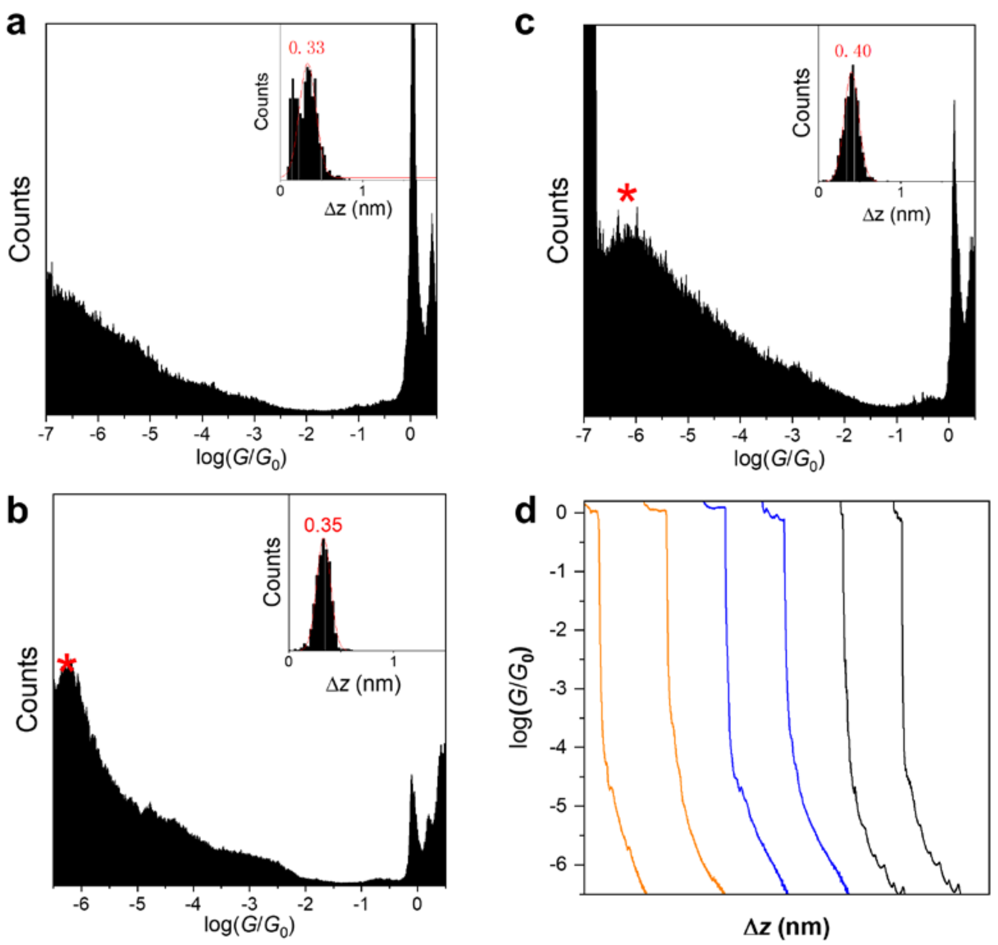

Supplementary Figure 10. **The blank experiment.** The one-dimensional histograms for pure solvents of **a** THF: TMB (1:4, *v*/*v*), **b** TCB and **c** *n*-decane. Insets: relative displacement distributions. Asterisks: back ground noise. **d** Typical individual traces in pure solvents THF/TMB (orange lines), *n*-decane (blue lines) and TCB (black lines).

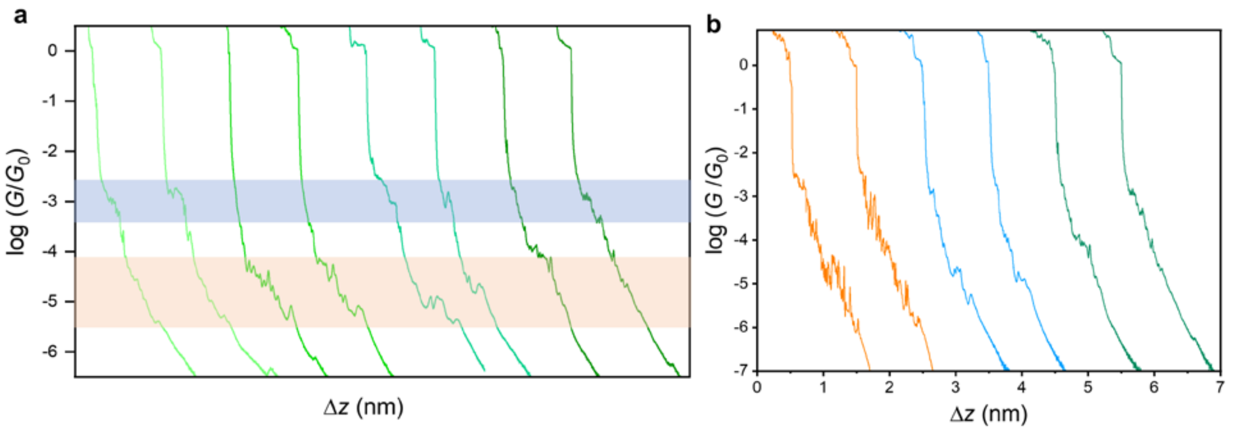

Supplemental Figure 11. Typical individual traces. a Typical individual traces for *o*-TP-2Py in THF: TMB (1:4, *v*/*v*). **b** Typical downhill individual traces for *o*-PP-2SMe (the orange lines), *o*-PP-2CN (the blue lines) and *o*-TP-2Py (the green lines) in THF: TMB (1:4, *v*/*v*).

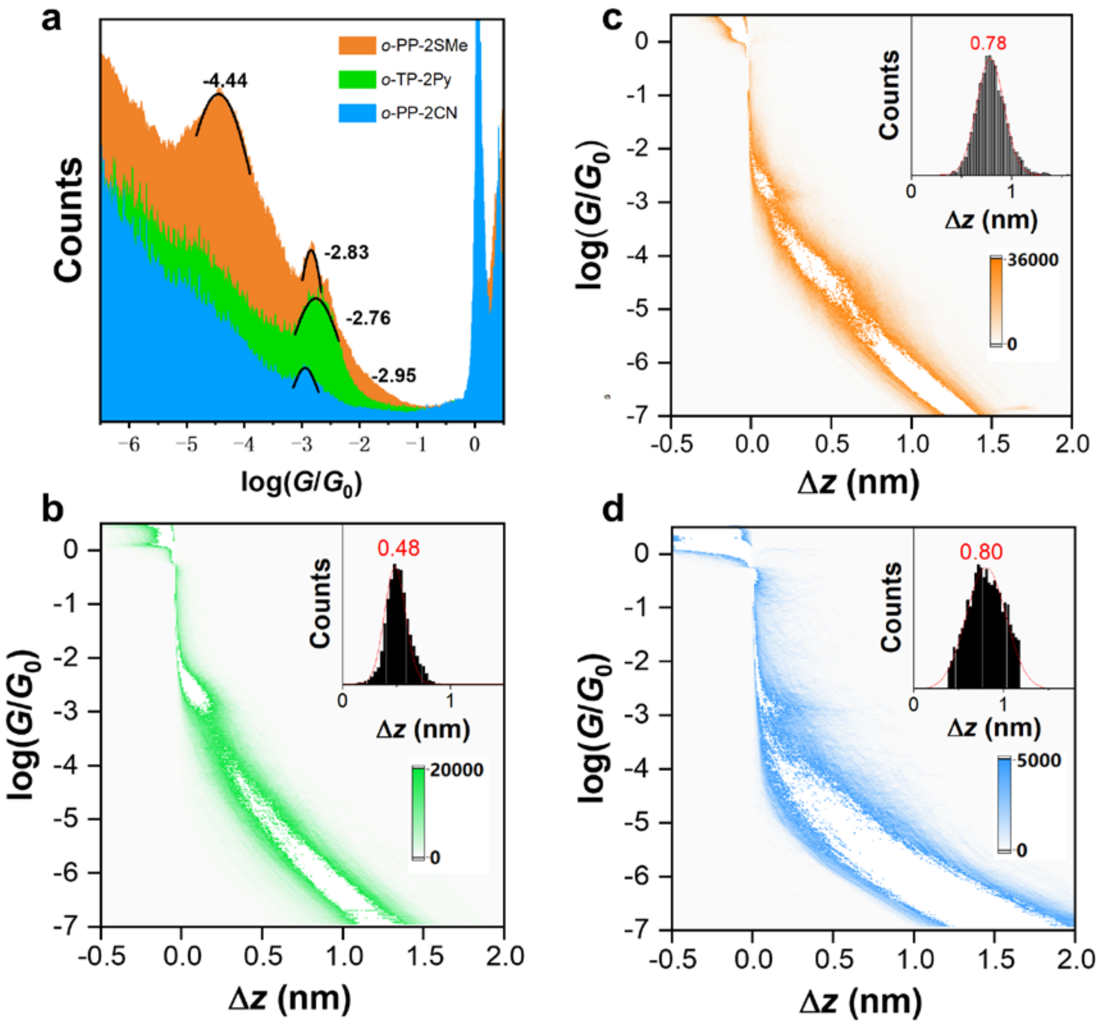

Supplementary Figure 12. **STM-BJ measurement of *o*-PP derivatives without data selection. a** One-dimensional histograms in THF: TMB (1:4, *v*/*v*) for *o*-PP-2SMe, *o*-TP-2Py and *o*-PP-2CN and two-dimensional histograms in THF: TMB (1:4, *v*/*v*) for **b** *o*-PP-2SMe, **c** *o*-TP-2Py and **d** *o*-PP-2CN without data selection. Insets: relative displacement distributions.

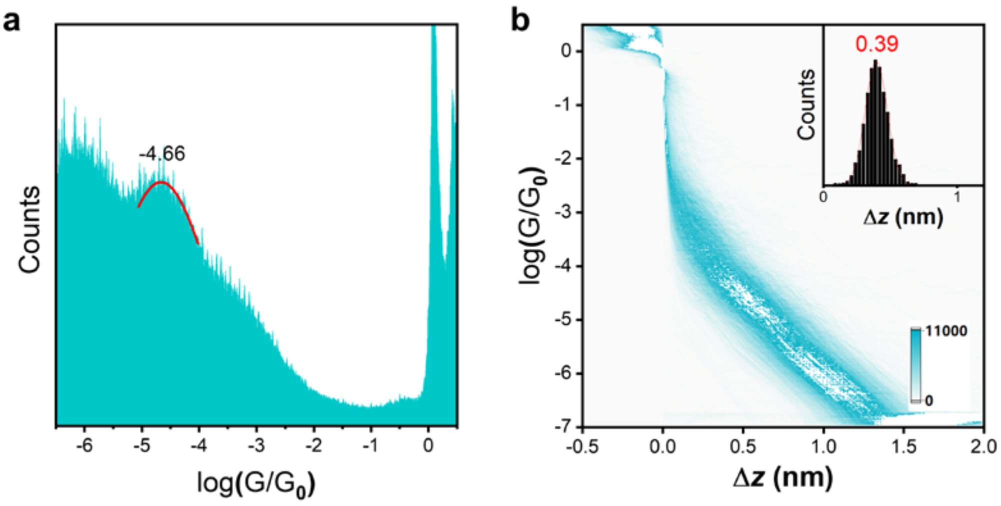

Supplementary Figure 13. STM-BJ measurement of *p*-PPy. **a** One-dimensional histogram and **b** two-dimensional histogram for *p*-PPy in THF: TMB (1:4, *v*/*v*).

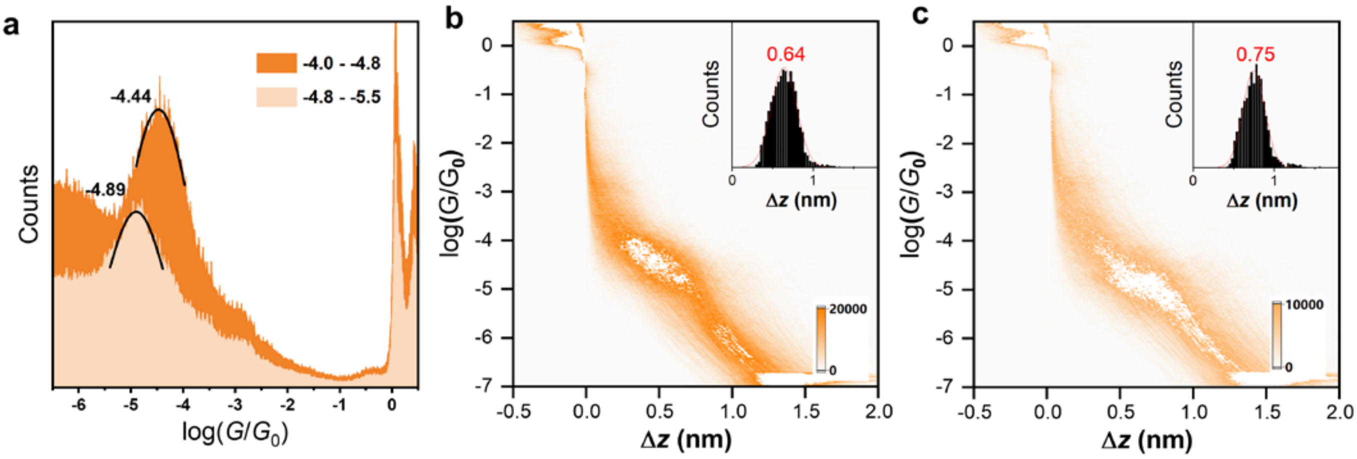

Supplementary Figure 14. **Conductance data selection of *o*-PP-2SMe. a** One-dimensional histograms for *o*-PP-2SMe with data selection among different conductance range in THF: TMB (1:4, *v*/*v*). **b** Two-dimensional histogram for *o*-PP-2SMe from $10^{-4.0}$ G_0 to $10^{-4.8}$ G_0 in THF: TMB (1:4, *v*/*v*). **c** Two-dimensional histogram for *o*-PP-2SMe from $10^{-4.8}$ G_0 to $10^{-5.5}$ G_0 in THF: TMB (1:4, *v*/*v*). Insets: relative displacement distributions.

Supplementary Table 2. Experimentally determined conductance of *o*-PP derivatives in different solvents.

	THF: TMB (1:4, v/v) (log(G/G_0))	*n*-decane (log(G/G_0))	TCB (log(G/G_0))
o-PP-2SMe	-2.83, -4.44, -4.89	-4.57, -4.93	-4.47, -4.90
o-PP-2CN	-2.95, -4.47	-4.97	–
o-TP-2Py	-2.73, -4.54	-4.94	–

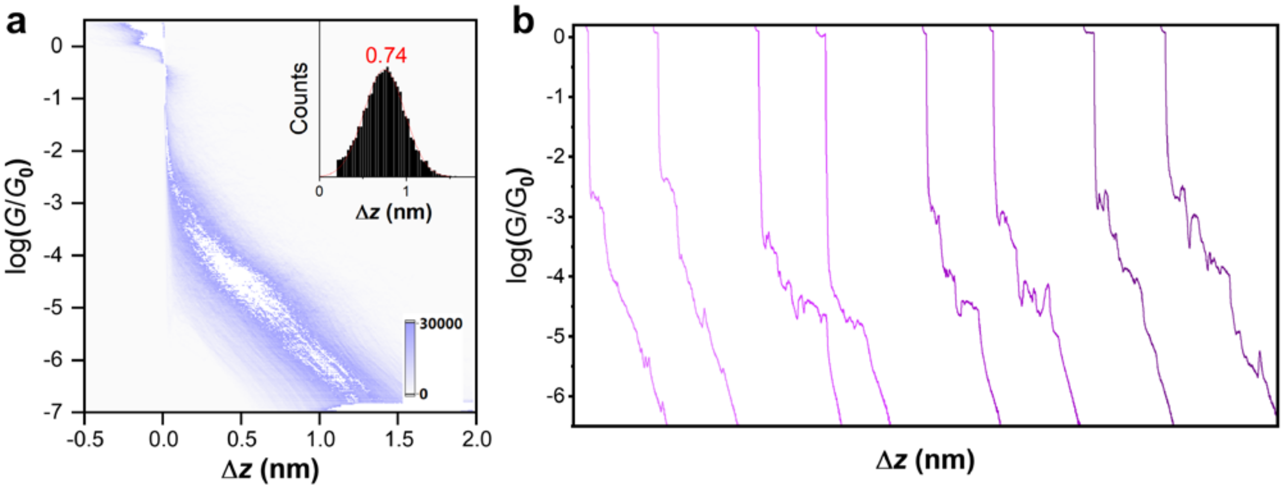

Supplementary Figure 15. (A) Two-dimensional conductance-displacement histogram for *o*-PP-2SMe in *n*-decane. Inset: relative displacement distributions. (B) Typical individual traces for *o*-PP-2SMe in *n*-decane.

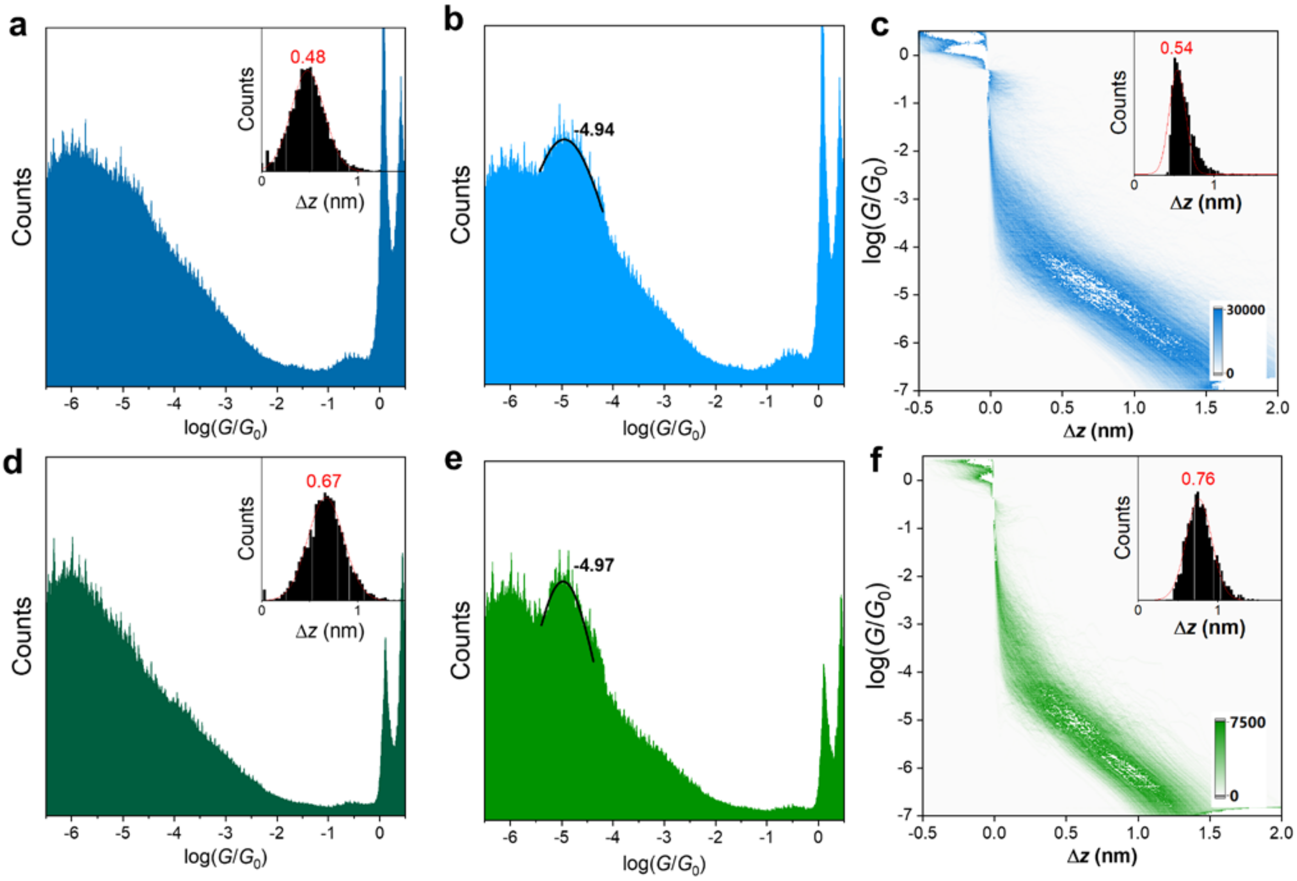

Supplementary Figure 16. **STM-BJ measurement of *o*-PP-2CN and *o*-TP-2Py.** One-dimensional histograms in *n*-decane for **a** *o*-TP-2Py and **d** *o*-PP-2CN without data selection. **b** One- and **c** two-dimensional histograms in *n*-decane for *o*-TP-2Py with data selection. **e** One- and **f** two-dimensional histograms in *n*-decane for *o*-PP-2CN with data selection. Insets: relative displacement distributions.

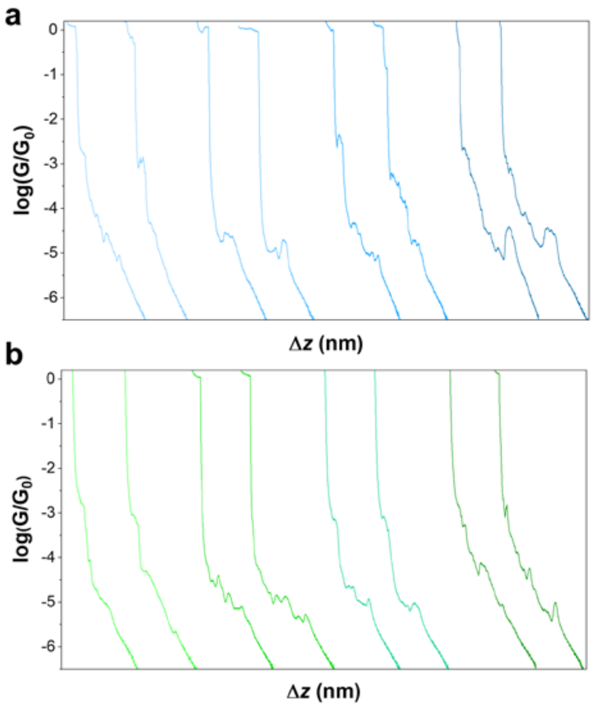

Supplementary Figure 17. Typical individual traces in *n*-decane of **a** *o*-TP-2Py and **b** *o*-PP-2CN.

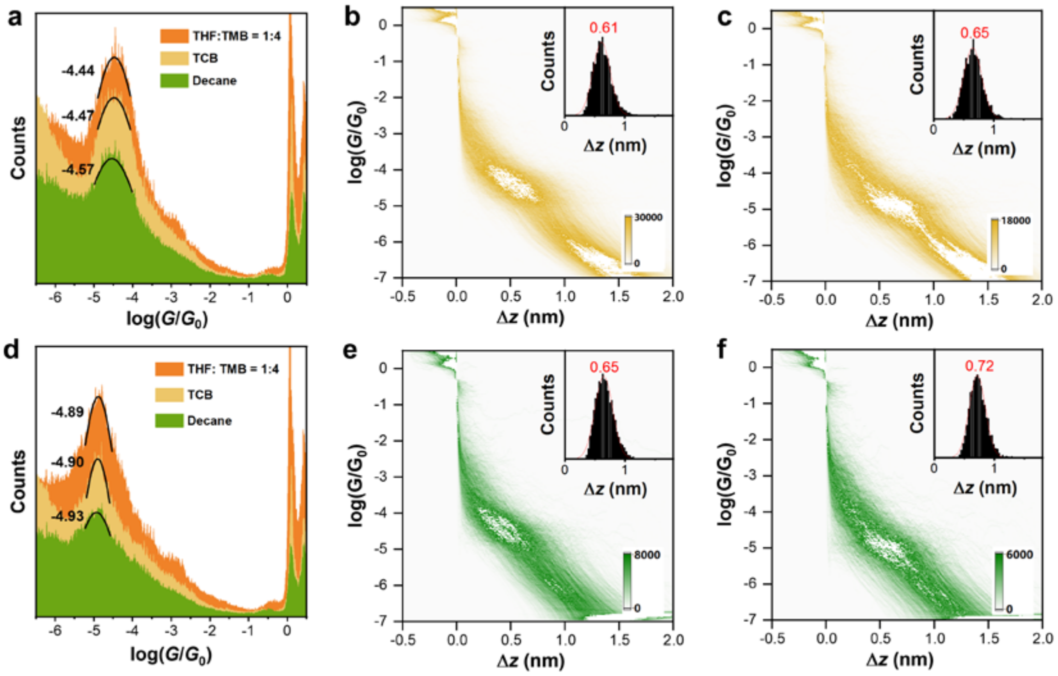

Supplementary Figure 18. Data selection of the conductance measurement for *o*-PP-2SMe in different solvents. a One-dimensional histograms for *o*-PP-2SMe from $10^{-4.0}$ G_0 to $10^{-4.8}$ G_0 in different solutions. **b** Two-dimensional histogram for *o*-PP-2SMe from $10^{-4.0}$ G_0 to $10^{-4.8}$ G_0 in TCB. **c** Two-dimensional histogram for *o*-PP-2SMe from $10^{-4.8}$ G_0 to $10^{-5.5}$ G_0 in TCB. **d** One-dimensional histograms for *o*-PP-2SMe from $10^{-4.8}$ G_0 to $10^{-5.5}$ G_0 in different solutions. **e** Two-dimensional histogram for *o*-PP-2SMe from $10^{-4.0}$ G_0 to $10^{-4.8}$ G_0 in *n*-decane. **f** Two-dimensional histogram for *o*-PP-2SMe from $10^{-4.8}$ G_0 to $10^{-5.5}$ G_0 in *n*-decane. Insets: relative displacement distributions.

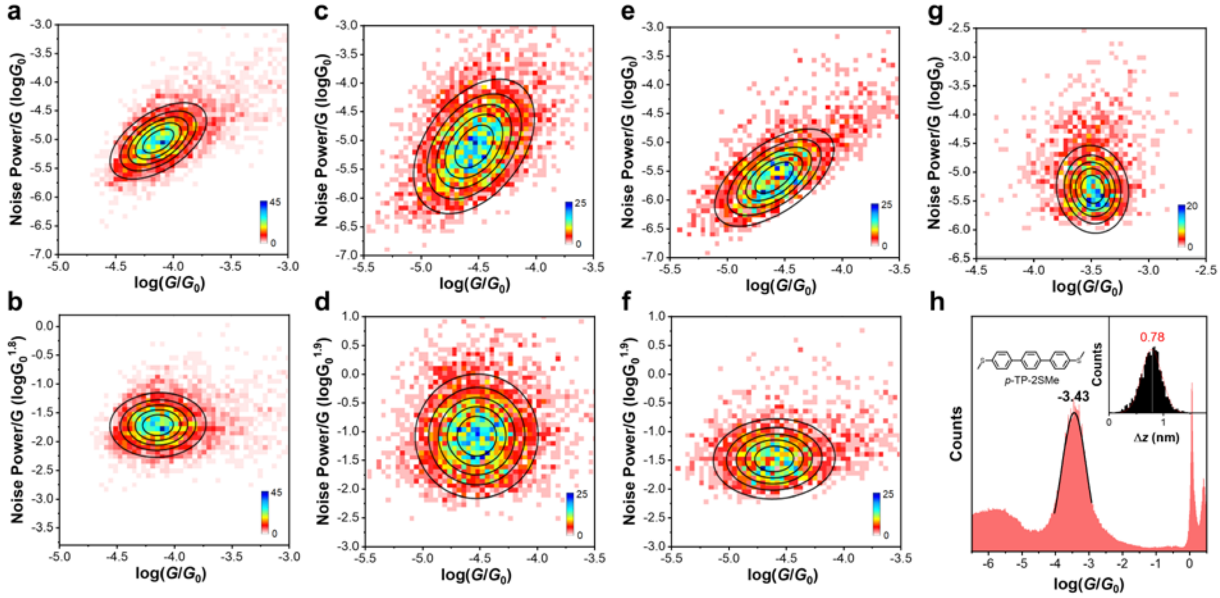

Supplementary Figure 19. **Flicker noise analyses of *o*-PP derivatives.** Two-dimensional histograms of normalized flicker noise power versus average conductance for **a** *o*-PP-2SMe, **c** *o*-PP-2CN and **e** *o*-TP-2Py. Two-dimensional histograms of flicker noise power normalized by G^n versus average conductance for **b** *o*-PP-2SMe (n = 1.8), **d** *o*-PP-2CN (n = 1.9), **e** *o*-TP-2Py (n = 1.9) and **g** *p*-TP-2SMe (n = 1.0). **h** One-dimensional histograms for *p*-TP-2SMe in THF : TMB (1:4, *v*/*v*).

Section 6. Theoretical calculation

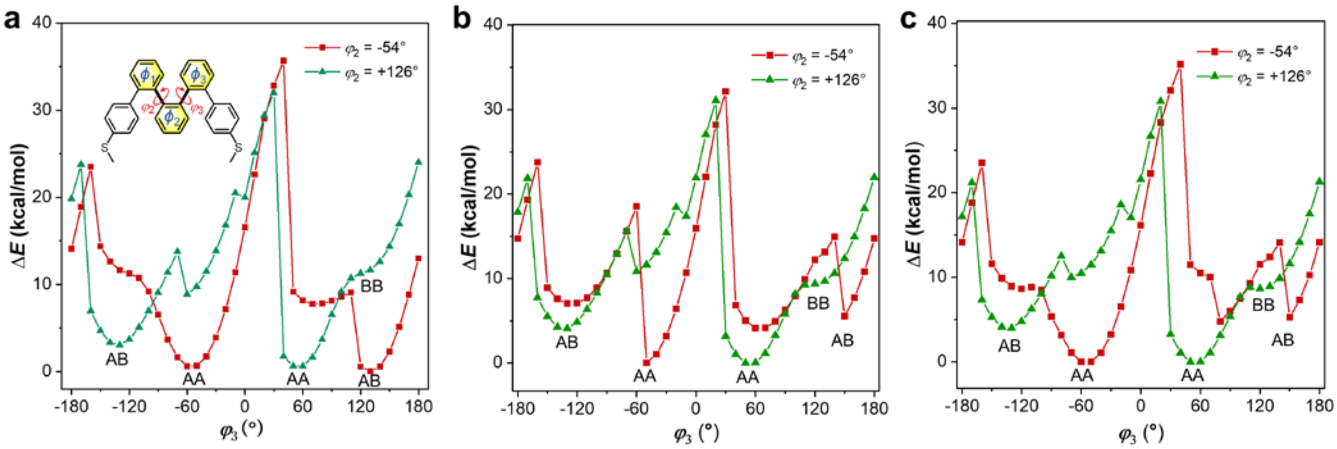

Supplementary Figure 20. Conformational energy profile for rotation about φ_3 with φ_2 held fixed at –54° or +126° of (A) *o*-PP-2SMe, (B) *o*-PP-2CN and (C) *o*-TP-2Py.

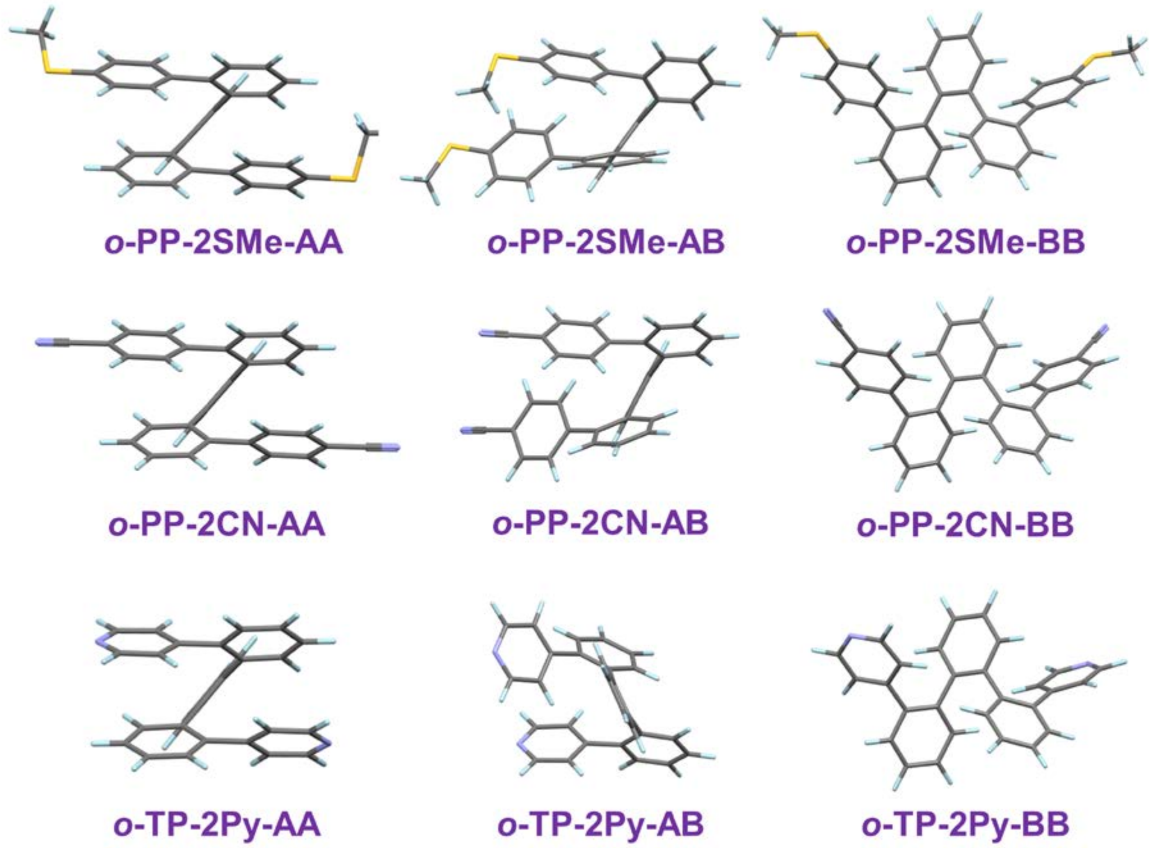

Supplementary Figure 21. Optimized geometries of stable conformers for *o*-TP-2Py and *o*-PP-2CN. The suffixes "AA", "AB" and "BB" denote AA, AB and BB conformers, respectively.

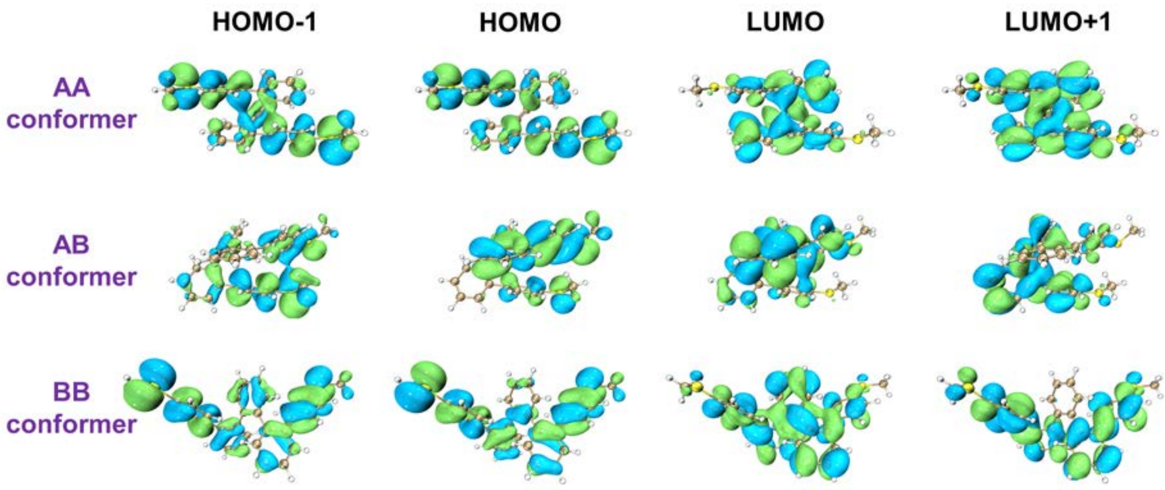

Supplementary Figure 22. Molecular orbitals for *o*-PP-2SMe.

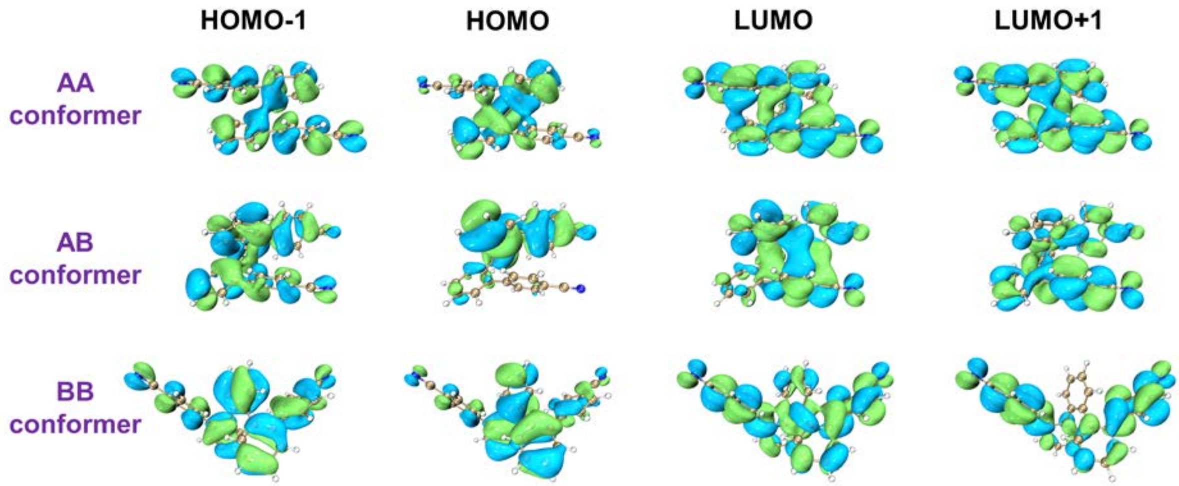

Supplementary Figure 23. Molecular orbitals for *o*-PP-2CN.

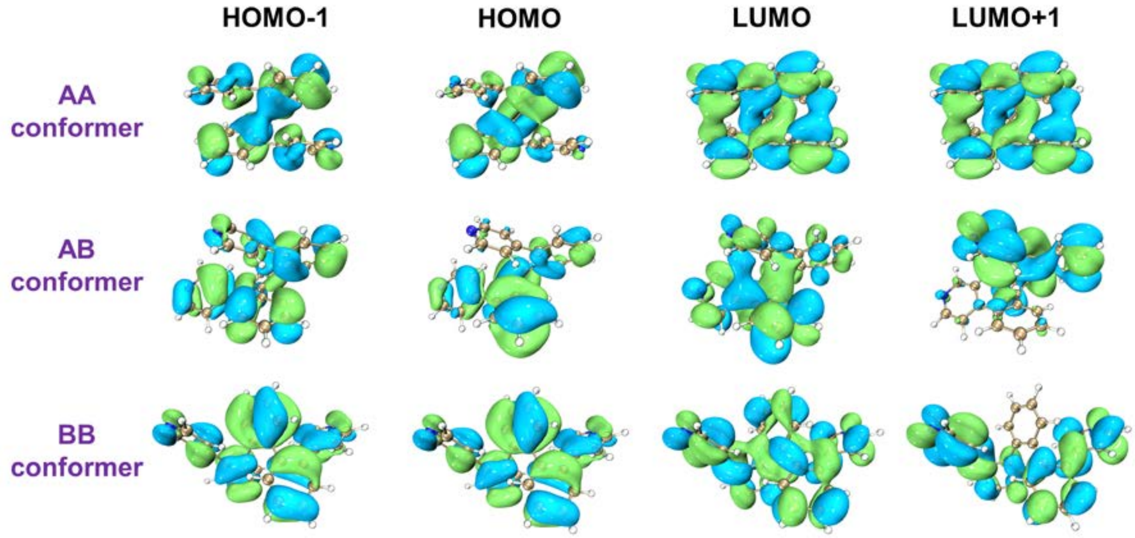

Supplementary Figure 24. Molecular orbitals for *o*-TP-2Py.

Supplementary Table 3. The calculated data of *o*-PP derivatives in different conformers.

	$L_{x\text{-}x}$[a] (Å)	ΔE_{THF}[b] (kcal/mol)	ΔE_{decane}[c] (kcal/mol)	μ_{THF} (D)	μ_{decane} (D)
***o*-PP-2SMe-AA**	12.21594	0	0	3.2735	2.7177
***o*-PP-2SMe-AB**	4.77488	1.9536	2.1090	3.6096	3.0382
***o*-PP-2CN-AA**	13.7507	0	0	0.3561	0.5680
***o*-PP-2CN-AB**	5.72531	2.1718	2.3475	5.6212	10.9450
***o*-TP-2Py-AA**	8.82064	0	0	0.3623	0.4875
***o*-TP-2Py-AB**	4.82659	2.0218	2.4239	6.4600	11.7992

[a] The optimized distances between two anchoring atoms. [b] The Gibbs free energy difference between the AA and AB conformers in THF. [c] The Gibbs free energy difference between the AA and AB conformers in THF. The suffixes “AA” and “AB” denote AA and AB conformers, respectively.

Supplementary Table 4. Calculated and experimentally determined single-molecule conductance of the new molecules in different conformers.[a]

Molecule	Cal. log(G/G_0)	Exp. log(G/G_0)
o-PP-2SMe-AA	-3.13	-4.44
o-PP-2SMe-AB	-1.65	-2.83
o-PP-2CN-AA	-3.51	-4.83
o-PP-2CN-AB	-1.33	-2.95
o-TP-2Py-AA	-3.71	-4.91
o-TP-2Py-AB	-1.41	-2.73

[a] The suffixes “AA” and “AB” denote AA and AB conformers, respectively.

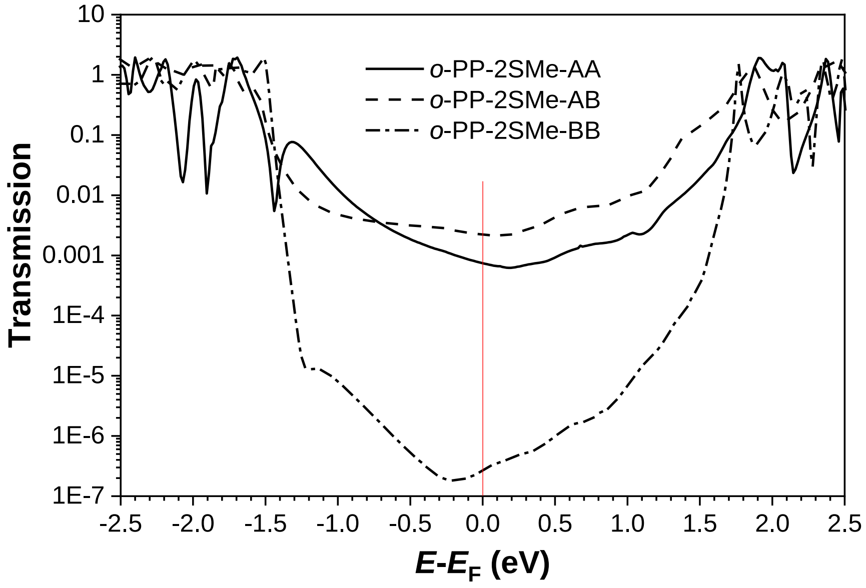

Supplementary Figure 25. The transmission spectra for *o*-PP-2SMe in different conformers. The suffixes “AA”, “AB” and “BB” denote AA, AB and BB conformers, respectively

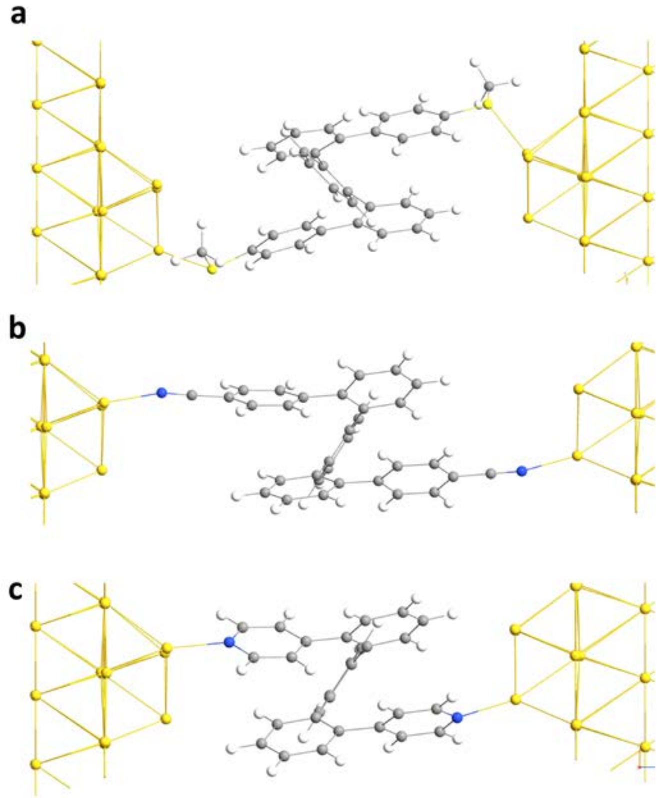

Supplementary Figure 26. **Device optimization.** The optimized device geometry for calculating the transmission coefficients of **a** *o*-PP-2SMe, **b** *o*-PP-2CN and **c** *o*-TP-2Py in AA conformers.

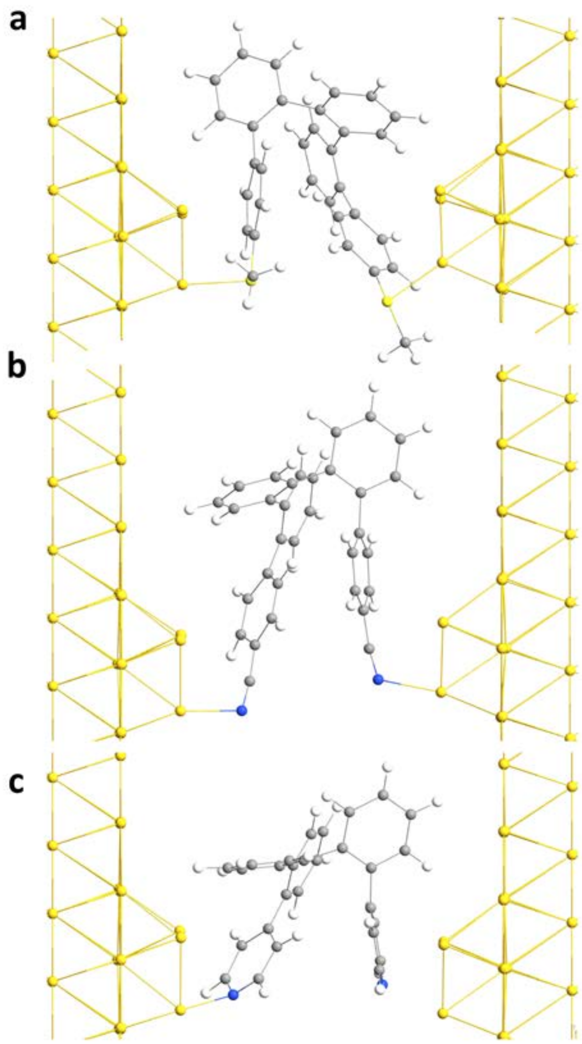

Supplementary Figure 27. **Device optimization.** The optimized device geometry for calculating the transmission coefficients of **a** *o*-PP-2SMe, **b** *o*-PP-2CN and **c** *o*-TP-2Py in AB conformers.

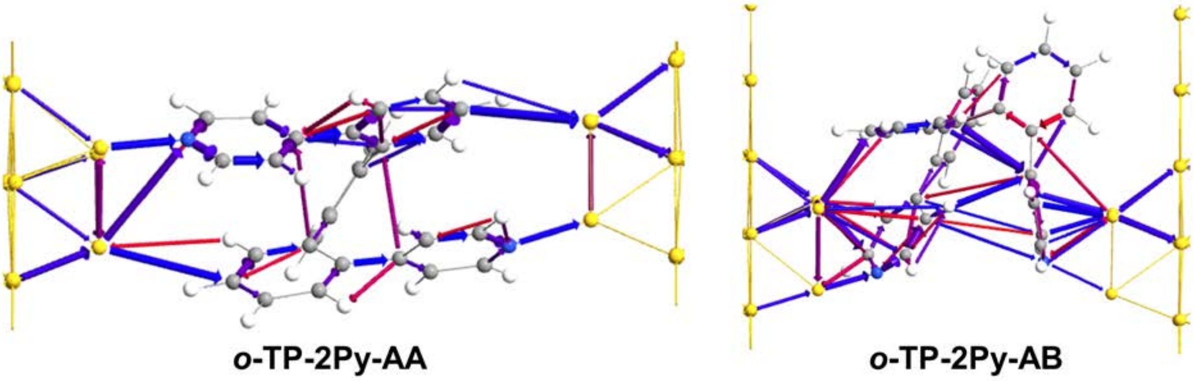

Supplementary Figure 28. **Transmission pathways for *o*-TP-2Py.** The suffixes “AA” and “AB” denote the AA and AB conformers, respectively. The blue arrows represent charge transport from the source electrode to the drain electrode and the red ones represent the transport in the opposite direction; the larger size of arrows also indicates the higher possibility of charge transport.

Section 7. NMR spectra

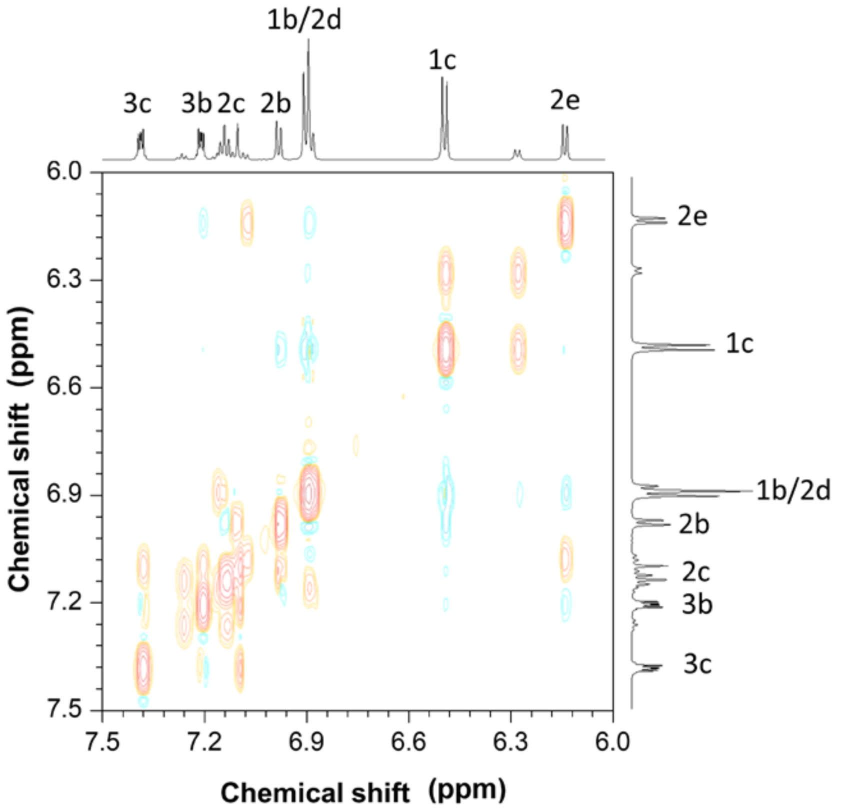

Supplementary Figure 29. EXSY NMR spectrum (500 MHz, CD_2Cl_2, −5 °C) for *o*-PP-2SMe.

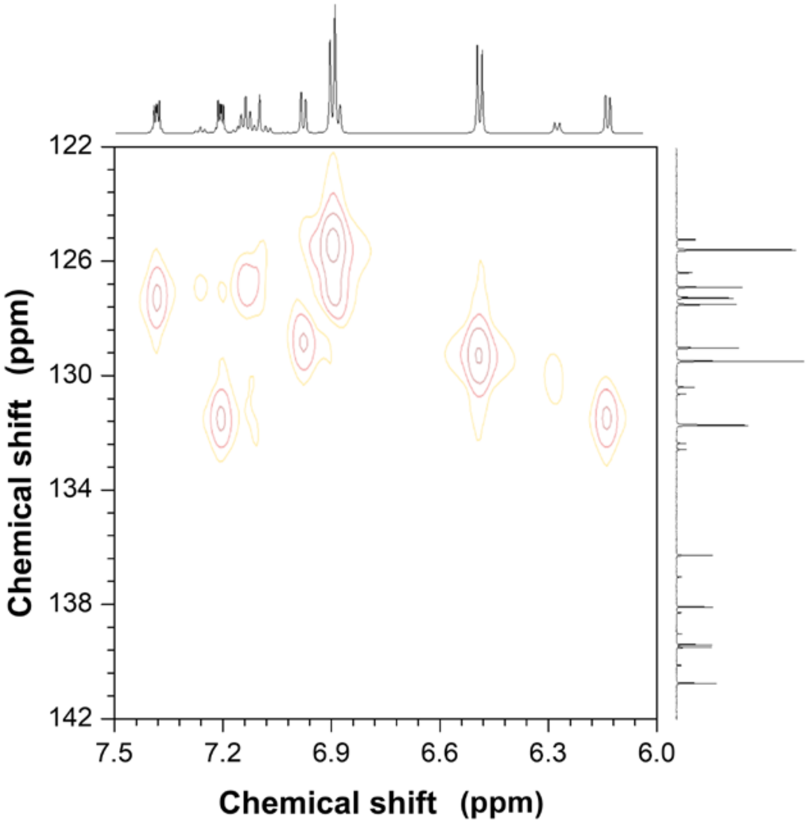

Supplementary Figure 30. HMQC NMR spectrum (500 MHz, CD_2Cl_2, −5 °C) for *o*-PP-2SMe.

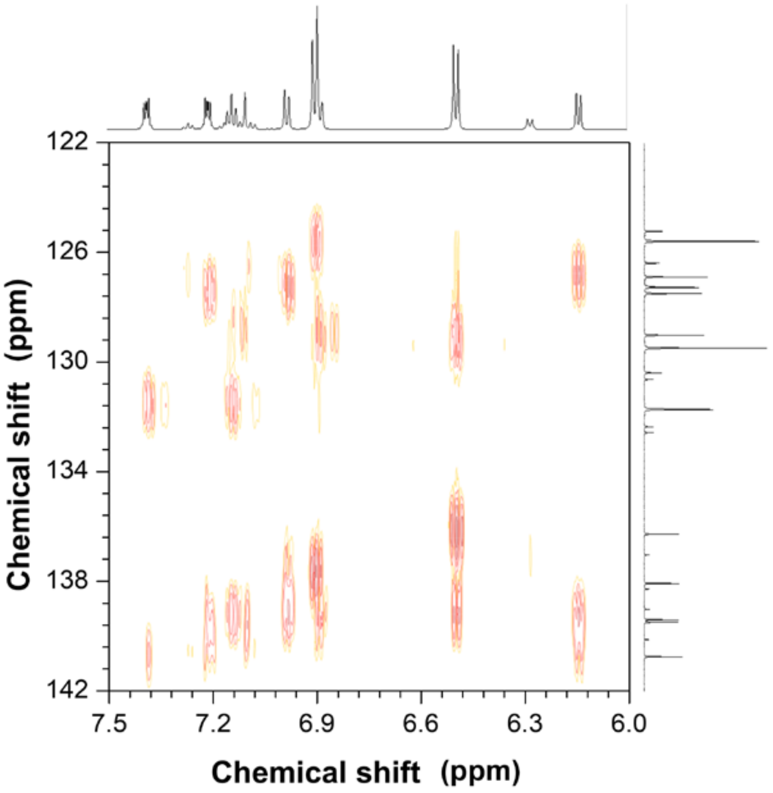

Supplementary Figure 31. HMBC NMR spectrum (500 MHz, CD_2Cl_2, –5 °C) of *o*-PP-2SMe.

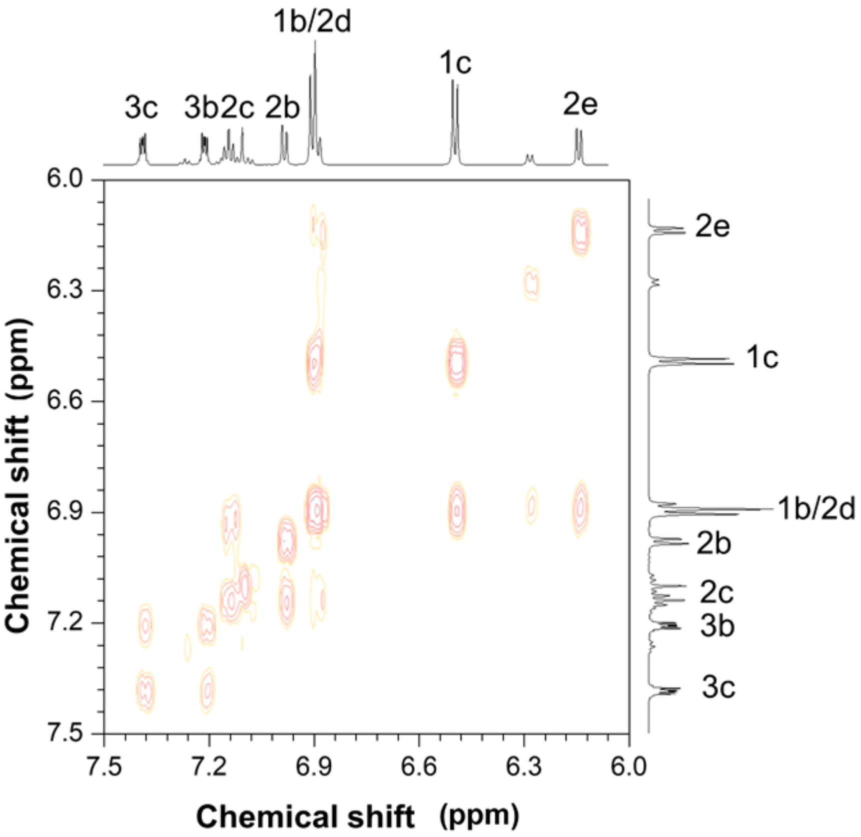

Supplementary Figure 32. COSY NMR spectrum (500 MHz, CD_2Cl_2, –5 °C) for *o*-PP-2SMe.

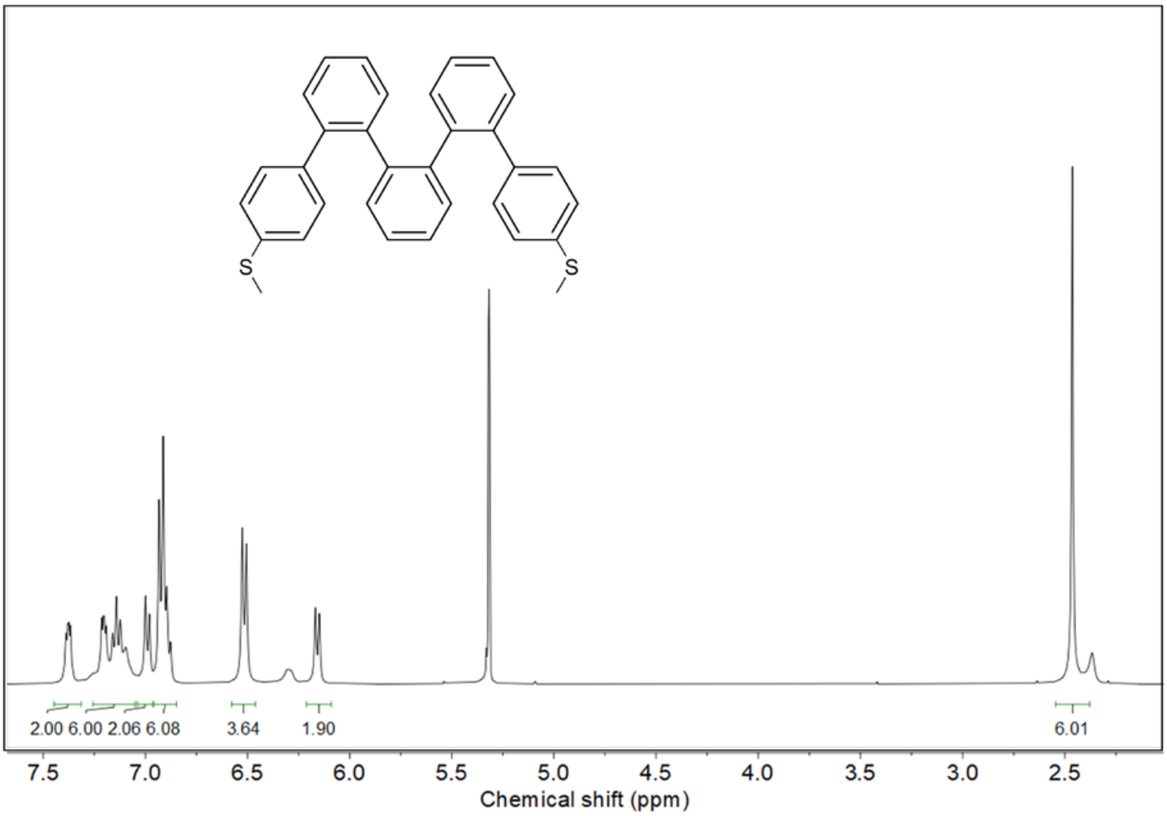

Supplementary Figure 33. ^{1}H NMR spectrum (500 MHz, CD_2Cl_2, room temperature) of *o*-PP-2SMe.

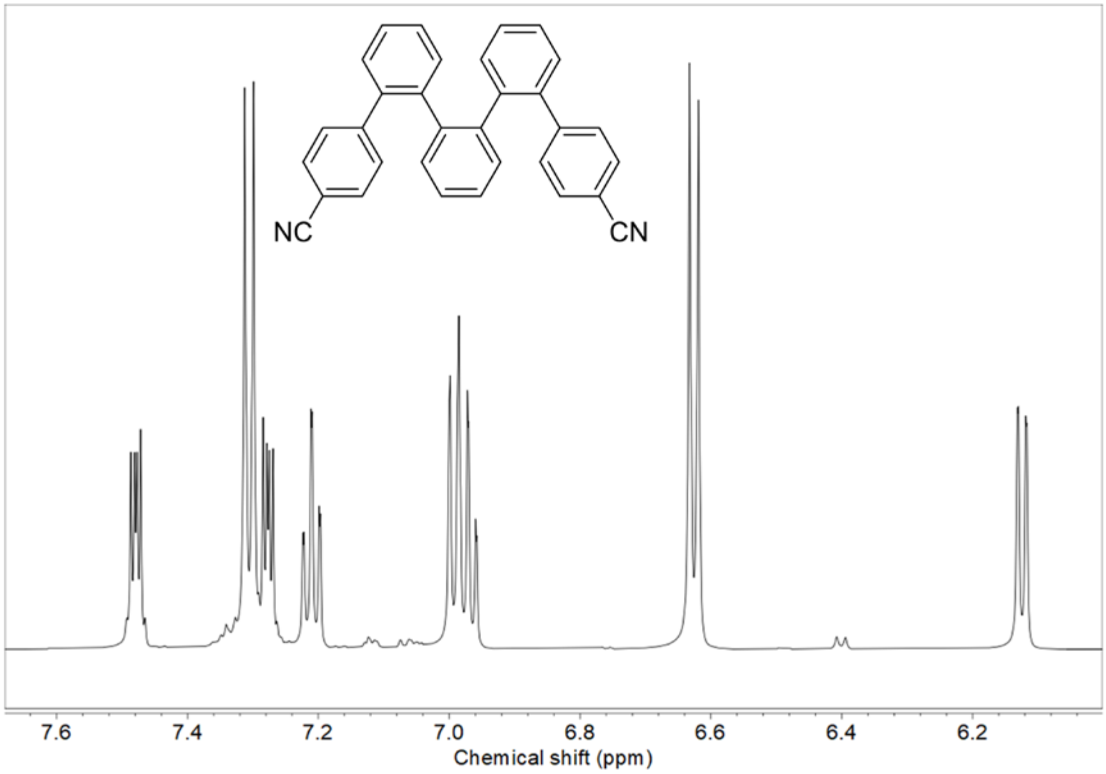

Supplementary Figure 34. ^{1}H NMR spectrum (500 MHz, CD_2Cl_2, -5 ℃) of *o*-PP-2CN.

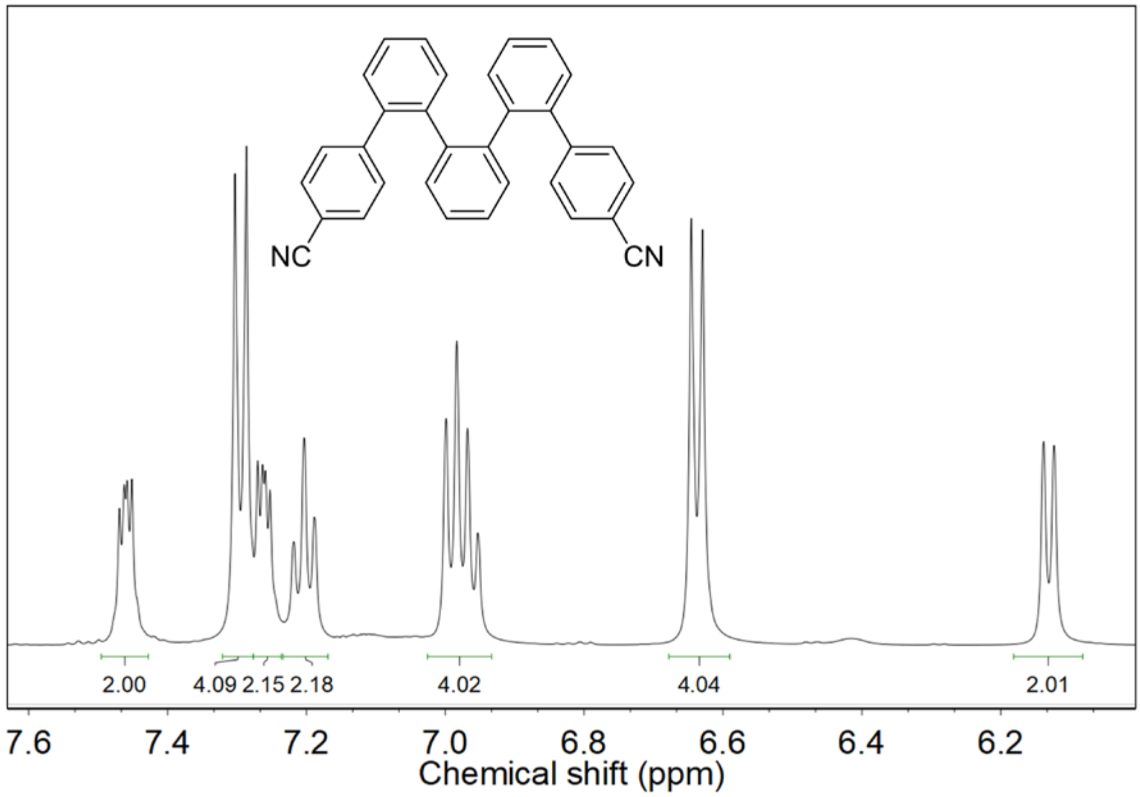

Supplementary Figure 35. ^{1}H NMR spectrum (500 MHz, CD_2Cl_2, room temperature) of *o*-PP-2CN.

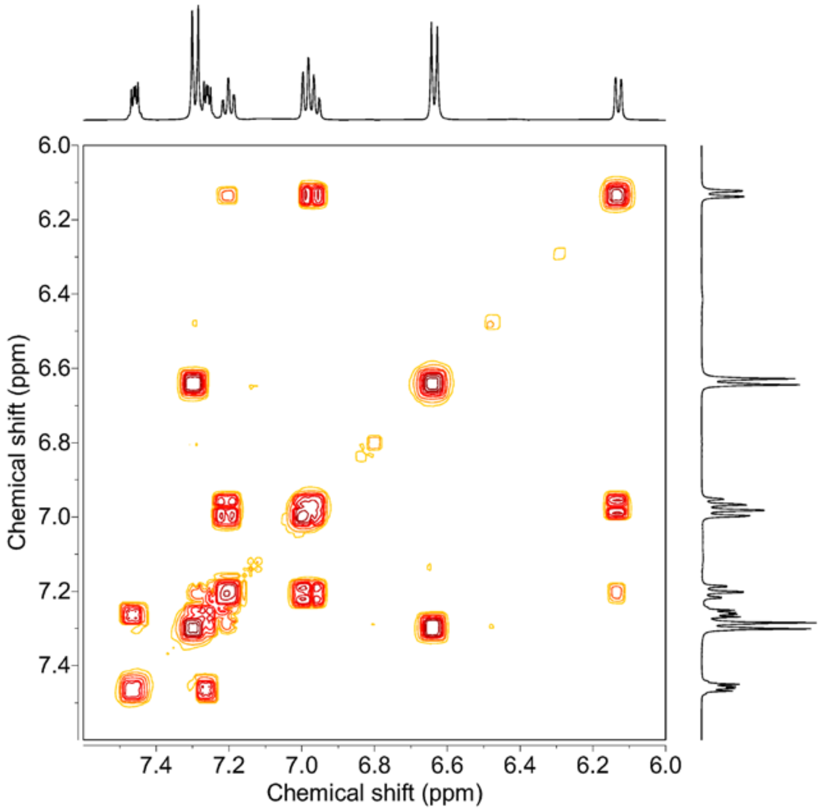

Supplementary Figure 36. COSY NMR spectrum (500 MHz, CD_2Cl_2, room temperature) for *o*-PP-2CN.

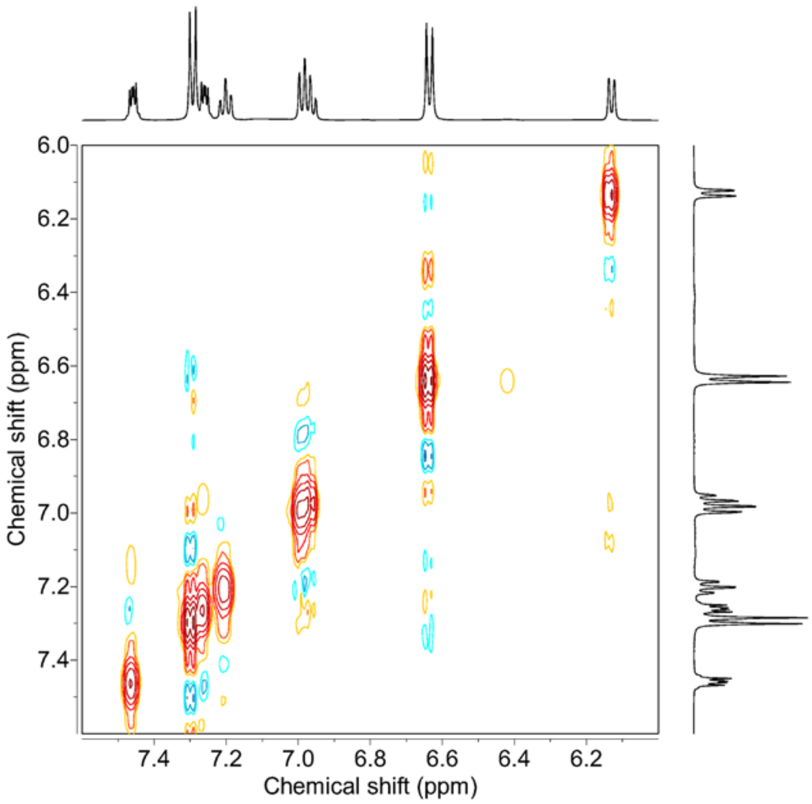

Supplementary Figure 37. EXSY NMR spectrum (500 MHz, CD_2Cl_2, room temperature) for *o*-PP-2CN.

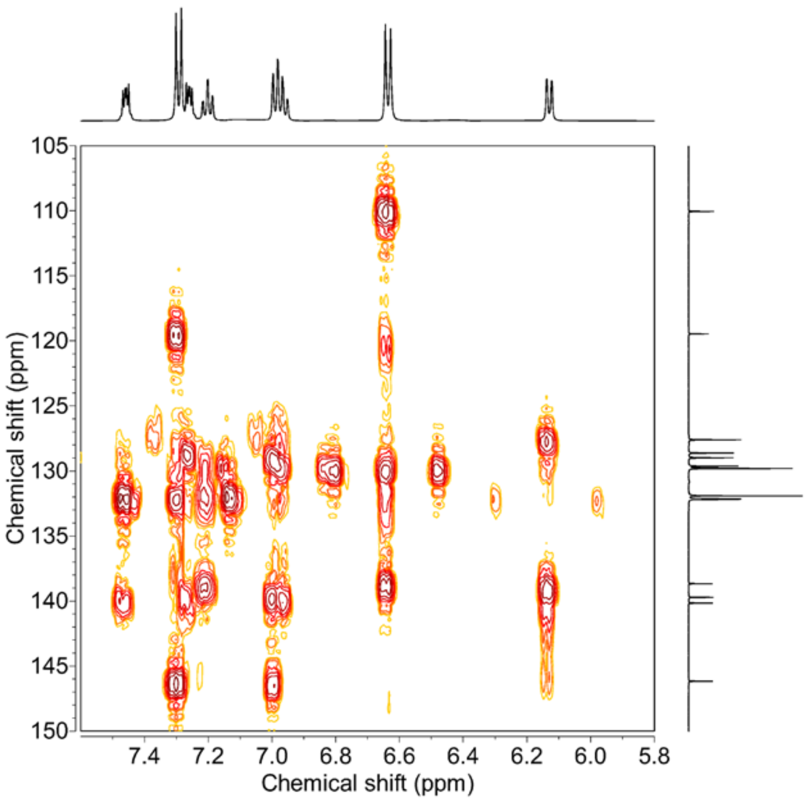

Supplementary Figure 38. HMBC NMR spectrum (500 MHz, CD_2Cl_2, room temperature) for *o*-PP-

2CN.

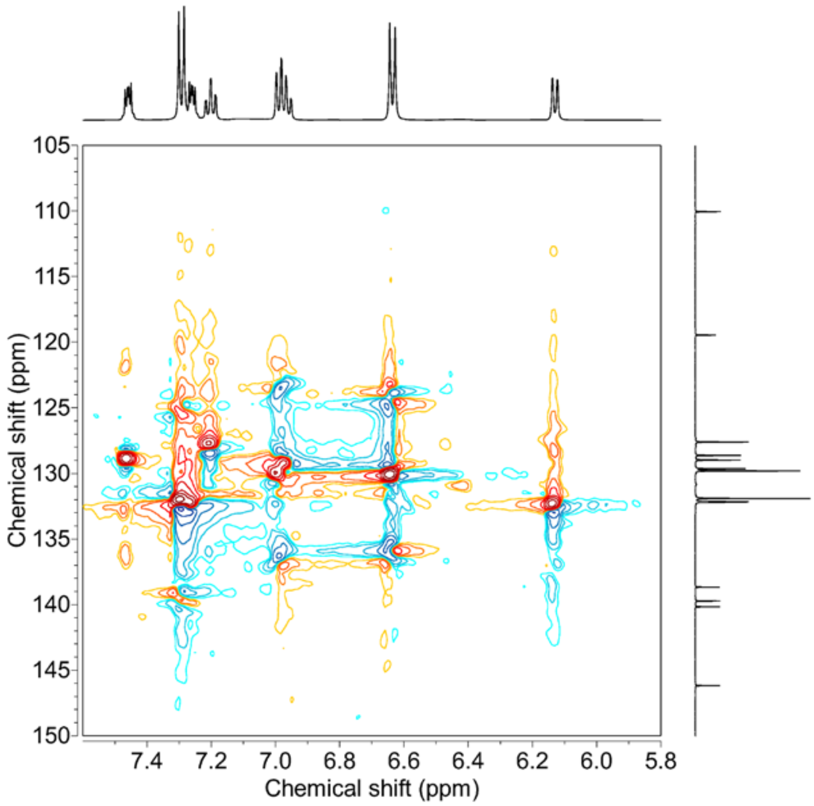

Supplementary Figure 39. HMQC NMR spectrum (500 MHz, CD_2Cl_2, room temperature) for *o*-PP-2CN.

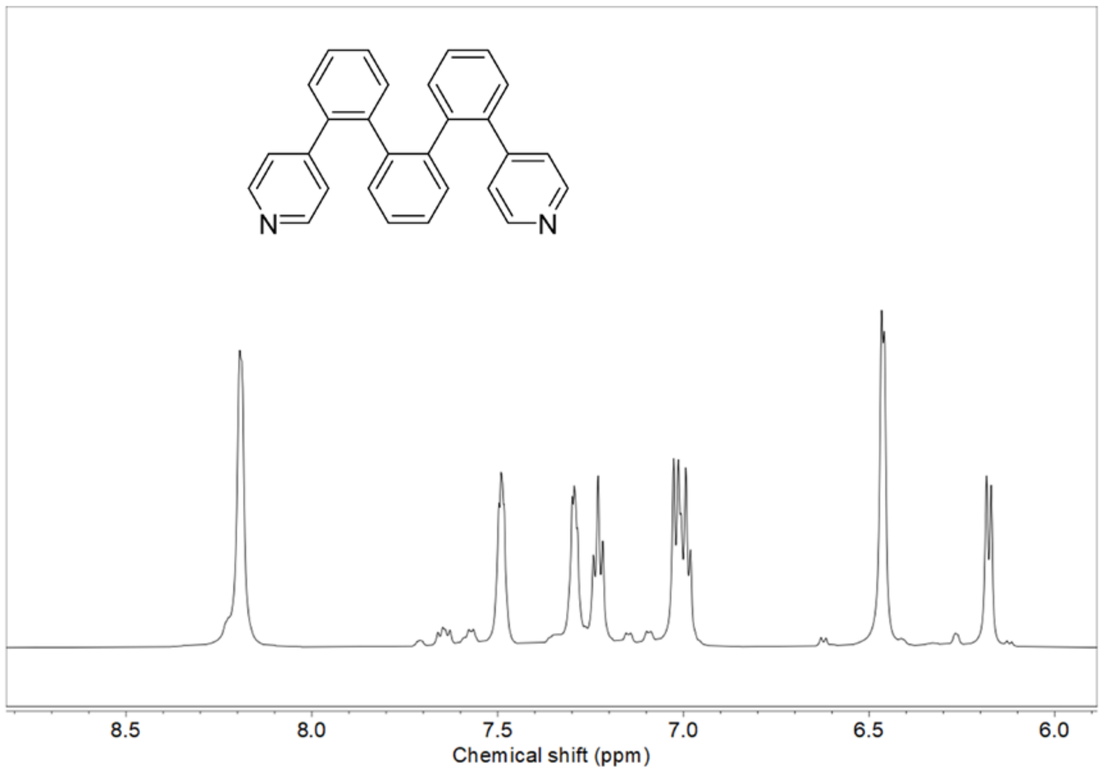

Supplementary Figure 40. ^{1}H NMR spectrum (500 MHz, CD_2Cl_2, -5 °C) of *o*-TP-2Py.

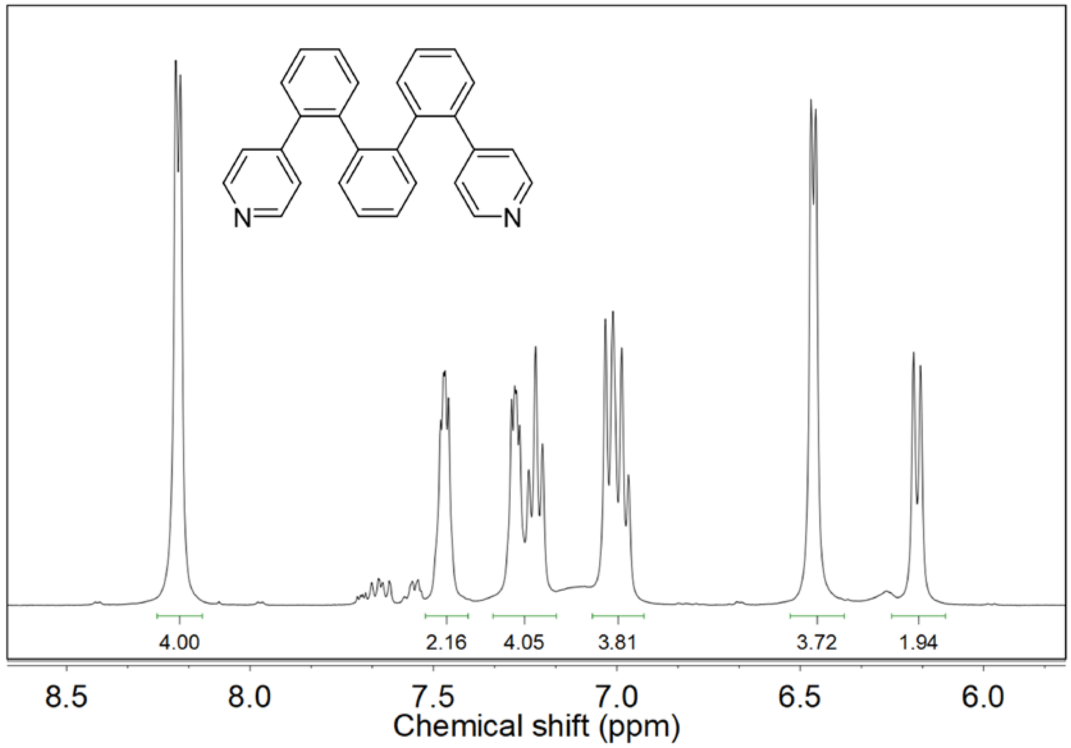

Supplementary Figure 41. ^{1}H NMR spectrum (500 MHz, CD_2Cl_2, room temperature) of *o*-TP-2Py.

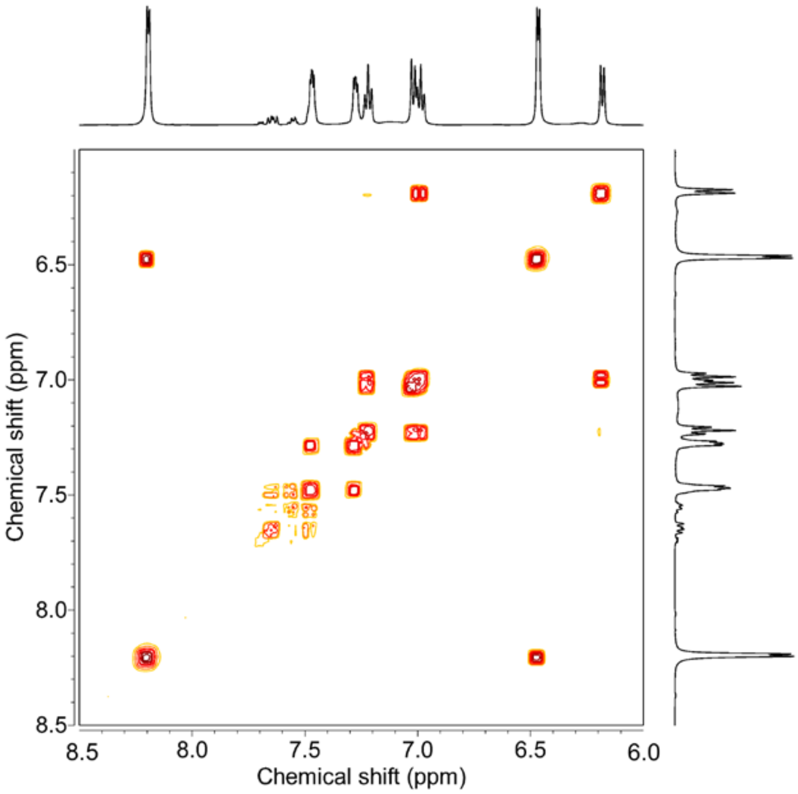

Supplementary Figure 42. COSY NMR spectrum (500 MHz, CD_2Cl_2, room temperature) for *o*-TP-2Py.

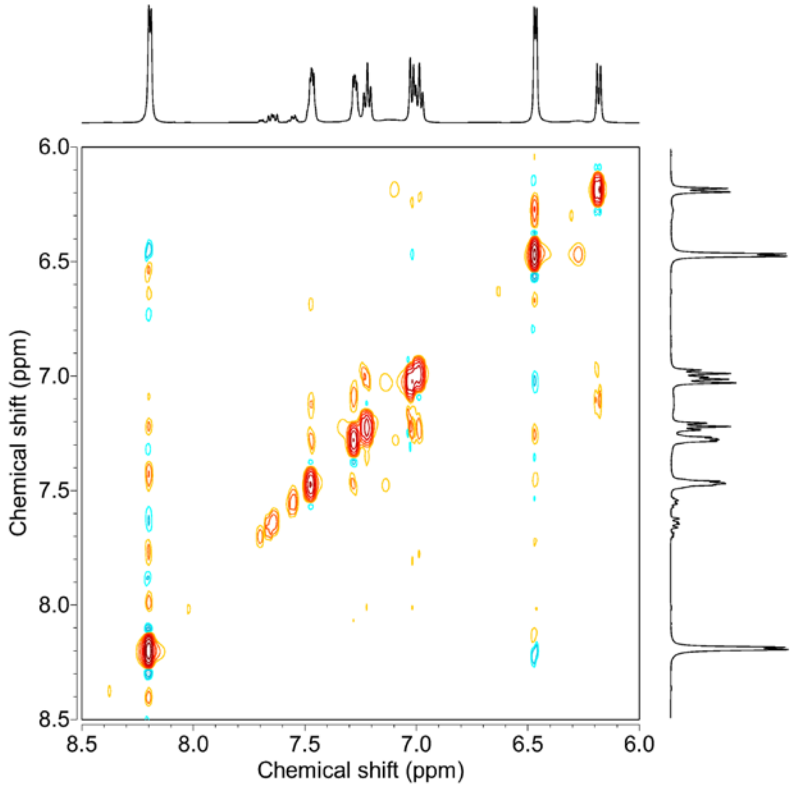

Supplementary Figure 43. EXSY NMR spectrum (500 MHz, CD_2Cl_2, room temperature) for *o*-TP-2Py.

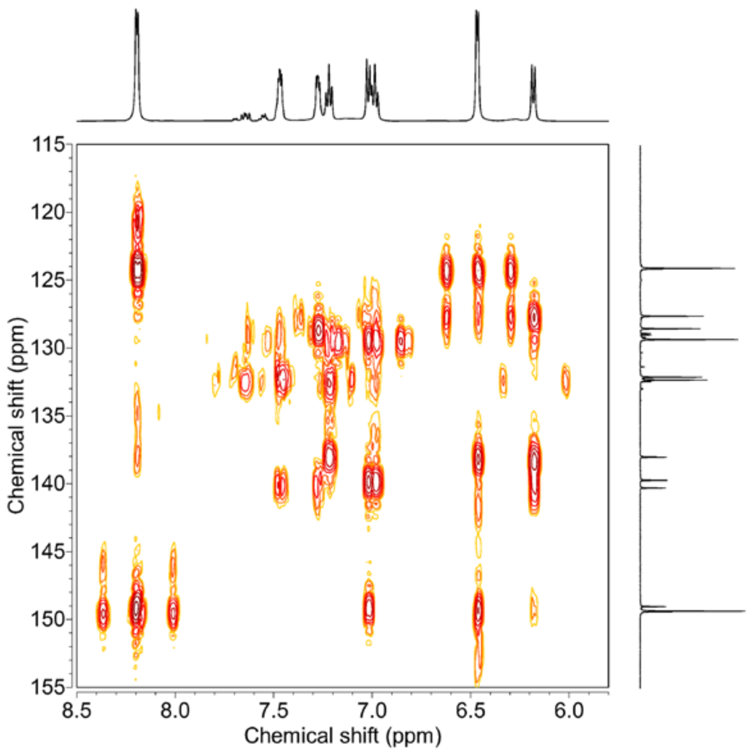

Supplementary Figure 44. HMBC NMR spectrum (500 MHz, CD_2Cl_2, room temperature) for *o*-TP-2Py.

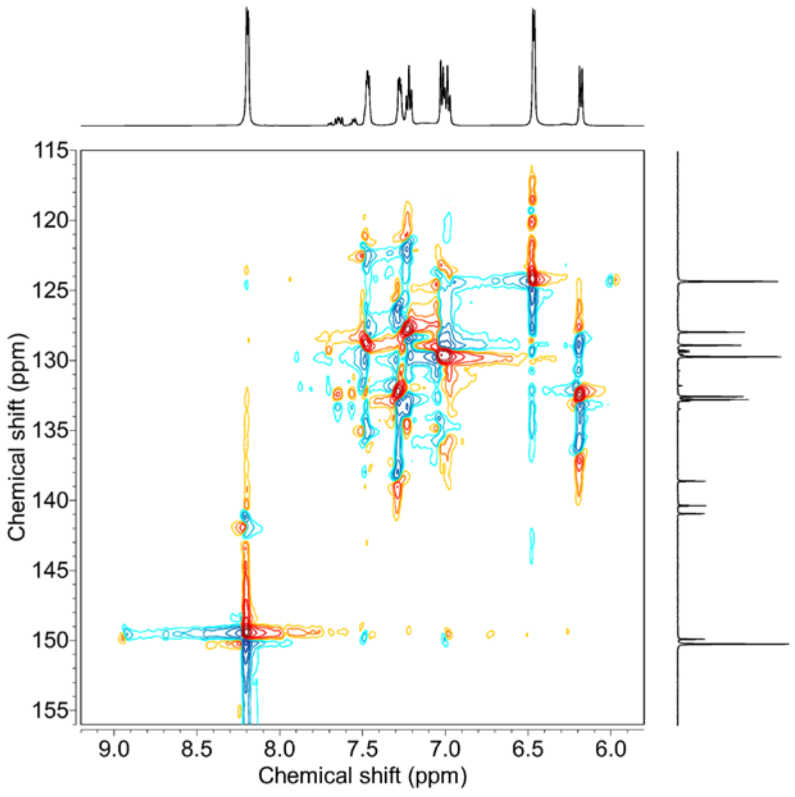

Supplementary Figure 45. HMQC NMR spectrum (500 MHz, CD_2Cl_2, room temperature) for *o*-TP-2Py.
